# Promising Anticancer Prodrugs Based on Pt(IV) Complexes with Bis-organosilane Ligands in Axial Positions

**DOI:** 10.1021/acs.jmedchem.3c02393

**Published:** 2024-04-09

**Authors:** Francisco Navas, Ana Chocarro-Calvo, Patricia Iglesias-Hernández, Paloma Fernández-García, Victoria Morales, José Manuel García-Martínez, Raúl Sanz, Antonio De la Vieja, Custodia García-Jiménez, Rafael A. García-Muñoz

**Affiliations:** † Group of Chemical and Environmental Engineering, Rey Juan Carlos University. C/Tulipán s/n, Móstoles, Madrid28933, Spain; ‡ Department of Basic Health Sciences. Rey Juan Carlos University. Avda. Atenas s/n, Alcorcón, Madrid 28922, Spain; § Endocrine Tumor Unit Chronic Disease Program (UFIEC). Carlos III Health Institute. Ctra. Majadahonda a Pozuelo km 2,2. Majadahonda, Madrid 28220, Spain

## Abstract

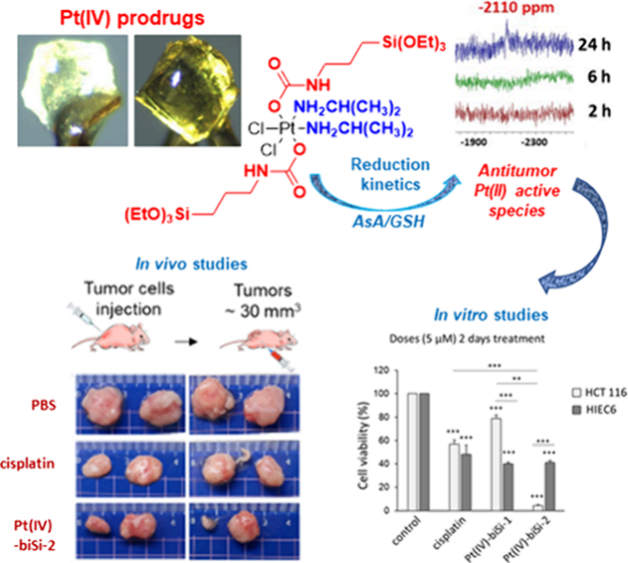

We report two novel prodrug Pt(IV) complexes with bis-organosilane ligands in axial positions: *cis*-dichloro(diamine)-*trans*-[3-(triethoxysilyl)propylcarbamate]platinum(IV) (Pt(IV)-biSi-1) and *cis*-dichloro(diisopropylamine)-*trans*-[3-(triethoxysilyl) propyl carbamate]platinum(IV) (Pt(IV)-biSi-2). Pt(IV)-biSi-2 demonstrated enhanced *in vitro* cytotoxicity against colon cancer cells (HCT 116 and HT-29) compared with cisplatin and Pt(IV)-biSi-1. Notably, Pt(IV)-biSi-2 exhibited higher cytotoxicity toward cancer cells and lower toxicity on nontumorigenic intestinal cells (HIEC6). In preclinical mouse models of colorectal cancer, Pt(IV)-biSi-2 outperformed cisplatin in reducing tumor growth at lower concentrations, with reduced side effects. Mechanistically, Pt(IV)-biSi-2 induced permanent DNA damage independent of p53 levels. DNA damage such as double-strand breaks marked by histone gH2Ax was permanent after treatment with Pt(IV)-biSi-2, in contrast to cisplatin's transient effects. Pt(IV)-biSi-2's faster reduction to Pt(II) species upon exposure to biological reductants supports its superior biological response. These findings unveil a novel strategy for designing Pt(IV) anticancer prodrugs with enhanced activity and specificity, offering therapeutic opportunities beyond conventional Pt drugs.

## Introduction

Cisplatin is the most widely used metallodrug in cancer chemotherapy and is highly effective in the treatment of solid cancers and hematological malignancies, increasing the cure rates from less than 10 to 85% in some tumors.^1^ This compound binds to DNA causing lesions that activate apoptotic pathways if they are not properly repaired.^2^ Alternatively, two other Pt(II) complexes have been approved for their use in cancer chemotherapy: carboplatin and oxaliplatin.^3^ However, Pt(II) complexes cause serious side effects such as ototoxicity, nephrotoxicity, hepatotoxicity, gastrointestinal disorders, hair loss, or anemia, which are a matter of concern.^4^ Intensive research into platinum derivatives that overcome these undesired effects has led to Pt(IV) complexes as among the most promising.^5,6^ These complexes present an octahedral geometry, instead of the typical square-planar disposition of the Pt(II) species, due to the oxidation process that introduces two extra ligands in axial positions of the metal. The saturation of the coordination sphere in Pt(IV) complexes drives resistance to ligand substitution reactions and prevents their inactivation by biomolecules present in the human body.^6^ An additional advantage of Pt(IV) complexes is their ease of modification through functionalization of axial groups to allow (i) better selectivity against tumor cells, (ii) improved cellular uptake, and (iii) better tolerance in biological media.^7^ The stability of Pt(IV) compounds makes the interaction rate and ligand exchange with DNA very slow.^8^ Therefore, Pt(IV) complexes are considered as prodrugs whose reduction inside the organism drives their biological activity.^9,11^ Two extra ligands in axial positions can enhance the solubility of Pt(IV) complexes, and their biological activity after oral administration has been tested in human clinical trials.^6^ This is the case for the complexes iproplatin (JM9), tetraplatin, and satraplatin (JM216). Iproplatin (JM9), extensively tested in phase II and III clinical trials, was definitively discarded because it was less cytotoxic than cisplatin and carboplatin.^10^ Tetraplatin entered phase I studies, but it was discarded because of serious neurotoxic side effects in patients.^11^ The antiproliferative activity of satraplatin (JM216) was studied in phase III trials, but it was abandoned because of the high variability in drug internalization.^12^ However, satraplatin is currently being tested in combination with other chemotherapeutic agents (docetaxel, paclitaxel, or capecitabine) in various tumor types.^10,13^

The canonical mechanism of reduction of Pt(IV) complexes involves the loss of the ligands in axial positions, rendering the divalent form capable of interacting with DNA and eventually eliciting the activation of apoptotic pathways.^6,14−16^ The rate of reduction is strongly dependent on the nature of the ligands bound to the Pt center, with the axial ligands exerting the stronger influence.^17,18^ Thus, based on the reduction potentials, Pt(IV) complexes with chloride axial ligands suffer faster reduction than carboxylate or hydroxo species.^19^ However, some Pt(IV) complexes with carbamate axial ligands show faster reduction than their carboxylate derivatives.^20^ Moreover, in the last years, some investigations have shown the formation of Pt(II) species in the reaction media that are different from those expected with the loss of the two axial ligands.^21^

The evaluation of the pharmacological properties of small molecules with silicon groups has received considerable attention in medicinal chemistry both *in vitro* and *in vivo*.^22,23^ The substitution of carbon by silicon in a molecular structure usually implies an increase in hydrophobicity,^23^ which may facilitate the transit through the plasma membrane to improve cellular uptake and increase the cytotoxic activity.^23,24^ Numerous studies have evaluated the cytotoxicity of organosilicon compounds against cancer cells *in vitro*. Recently, two patented disiloxanes (SILA-409 and SILA-421) have proven to be effective in reversing multidrug resistance (MDR) in some human colorectal adenocarcinoma cell lines. Both organodisiloxane compounds, at very low doses, decreased the resistance of colorectal cancer cells to doxorubicin.^25^ Another organosilicon compound, GH1504, inhibited the growth of prostate cancer cells *in vitro* and *in vivo* and demonstrated *in vitro* antiproliferative properties in a wide panel of human cancer cells.^26^ The mechanisms through which these complexes target cell death in tumors remain unclear.^23,26^

Herein, we report the synthesis of two Pt(IV) complexes derived from classical *cis*-Pt(II) complexes. These complexes feature aliphatic amines and chloride ligands in the equatorial plane accompanied by the introduction of two bis-organosilane moieties in axial positions. This design choice offers a unique advantage, allowing us to tune and modify the nature of the axial ligands. This work evaluates the influence of these axial ligands on the antitumor activity and selectivity of such compounds *in vitro* using malignant and healthy human intestinal cells and *in vivo* in a preclinical mouse model of colorectal cancer. Our results reveal differences in the mechanisms underlying the cytotoxicity of cisplatin versus Pt(IV) complexes and also between different Pt(IV) complexes. ^195^Pt NMR monitoring of the generation of active Pt(II) species over time upon reduction by biological reducing agents reveals differences that may explain the differential toxicity of Pt(IV) complexes.

## Results and Discussion

### Synthesis and Characterization of the Platinum Complexes

**Figure 1:**
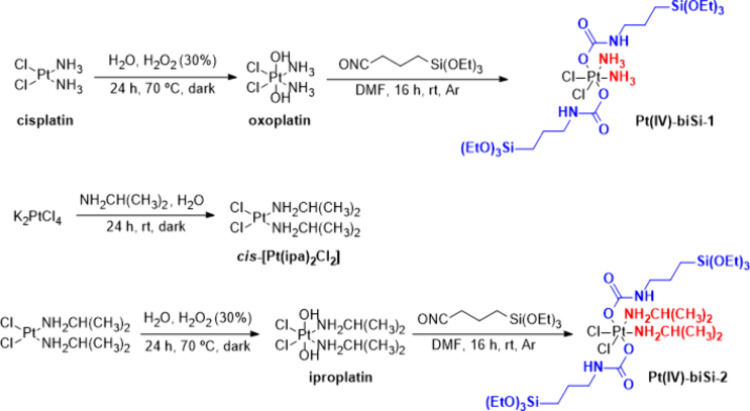
Synthetic routes to obtain platinum complexes Pt(IV)-biSi-1 and Pt(IV)-biSi-2. In both Pt(IV) final complexes, the bis-organosilane ligands are colored blue and the amine moieties red.

We first put all the efforts into developing the synthesis of Pt(IV) complexes with carbamate moieties and terminal siloxane groups in the axial positions. Figure 1 shows the overall synthesis to obtain the final complexes Pt(IV)-biSi-1 and Pt(IV)-biSi-2. The synthesis started from the *cis*-Pt(II) precursors (cisplatin and *cis*-[Pt(ipa)_2_Cl_2_]), which are oxidized to the Pt(IV) intermediates with H_2_O_2_, with the introduction of two hydroxyl groups in the axial positions (thus generating oxoplatin and iproplatin). Finally, the subsequent functionalization of these −OH groups by the formation of the carbamate groups affords the formation of the final Pt(IV) complexes with bis-organosilane ligands Pt(IV)-biSi-1 and Pt(IV)-biSi-2.

**Figure 2:**
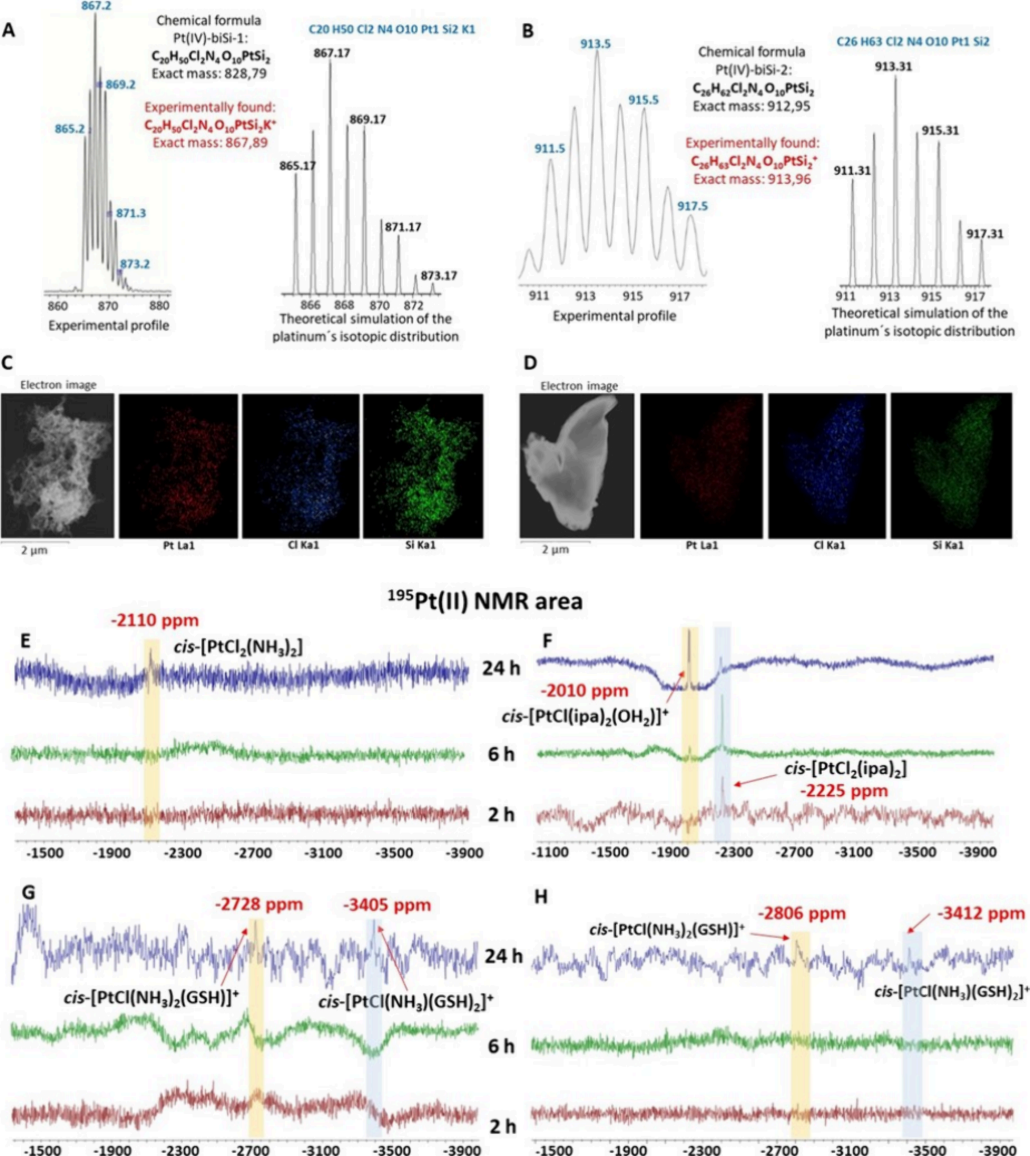
ESI-MS spectra (positive mode) of (A) Pt(IV)-biSi-1 and (B) Pt(IV)-biSi-2. STEM/EDX mapping analysis of the morphology and element distribution (platinum, chloride, and silicon) on the surface of (C) Pt(IV)-biSi-1 and (D) Pt(IV)-biSi-2. ^195^Pt NMR spectra in the Pt(II) region of the reduction kinetics at different times of (E) Pt(IV)-biSi-1 and AsA, (F) Pt(IV)-biSi-2 and AsA, (G) Pt(IV)-biSi-1 and GSH, and (H) Pt(IV)-biSi-2 and GSH.

The complex *cis*-[Pt(ipa)_2_Cl_2_], the precursor of the Pt(IV) intermediate iproplatin, was generated from the reaction between K_2_PtCl_4_ salt and isopropylamine (ipa) in a well-known one-step ligand substitution reaction.^27^ Analyzing the ^1^H NMR spectrum of iproplatin (Figure S2) with regard to its Pt(II) counterpart (Figure S1), it can be observed that the corresponding signals of the aliphatic protons close to the metal center have suffered a clear deshielding due to the oxidation process (see Table S1). Moreover, the signals obtained in the ^195^Pt NMR spectra of iproplatin and oxoplatin (Figures S2 and S4, respectively) agree with the values reported previously,^28^ confirming that the oxidation process with H_2_O_2_ was successful in both cases. Iproplatin and oxoplatin were also characterized by FTIR (Figures S3 and S5), and the obtained spectra also are also in good agreement with those previously reported.^28^ The most characteristic band in both cases is the Pt–OH bend, which appears at around 1030–1050 cm^–1^. It is important to note that, in the case of oxoplatin, the synthesis yielded yellow crystals that needed to be washed with water to eliminate the perhydrate form, caused by the presence of H_2_O_2_ in excess, which showed an intense band at ν(−OH) at 3417 cm^–1^. When the perhydrate was completely removed from the coordination sphere, this band disappeared, forming a new band of ν(−OH) at 3520 cm^–1^. This perhydrate form was not observed in the case of iproplatin. The formation of the bis-organosilane Pt(IV) complexes was achieved by subsequent functionalization of the −OH ligands. The reaction between these ligands and the isocyanate group of the organosilylated molecule affords the desired complexes with a carbamate group in each axial position (Figure 1). Although Pt(IV) complexes with carbamate groups in the axial positions have been previously synthesized using different methodology,^29^ the novelty of the herein used approach resides in the fact that these complexes have terminal siloxane groups in the axial positions. Here, the use of an additional solvent such as DMF allows the formation of the complexes using only a small excess of the corresponding isocyanate, as it had already been described by Wilson and Lippard.^30^ The ^1^H NMR spectra of both compounds show the corresponding signals of the silylated and aliphatic fragments of the new axial ligands in the expected range (Figures S6 and S9). The formation of the carbamate groups resulted in the appearance of two signals assigned to the −OCONH– proton (6.54 and 5.93 for Pt(IV)-biSi-1 and 6.84 and 6.52 for Pt(IV)-biSi-2) due to the presence of two configurational isomers, which had been previously reported.^20,30^ Obviously, the more intense signal corresponds to the major isomer and the less intense signal to the minor isomer. In the ^195^Pt NMR spectra of both complexes, two signals are also identified for each complex, confirming again the presence of the two isomers. These signals in both ^195^Pt NMR spectra are in the expected range of 1250–1370 ppm (Figures S6 and S9), confirming the formation of the *c-t-c-*Pt(IV)N_2_(OCONH)_2_Cl_2_ moiety, also in agreement with other previous studies.^20,30−33^ The high sensitivity of the ^195^Pt NMR technique is confirmed through the influence exerted by carbamate groups on the Pt center when analyzing the clear deshielding produced in the signals of both Pt(IV) complexes with respect to their Pt(IV) precursors. In these complexes, the nature of the axial and equatorial ligands also has a direct effect on the chemical shift of the −NH groups of the amine moieties bound to the platinum atom.^34^ For this reason, the −NH_3_ groups in the equatorial positions of Pt(IV)-biSi-1 have a chemical shift of 6.68 ppm in the ^1^H NMR spectrum, which is in agreement with other previously reported complexes.^35^ On the other hand, in the ^1^H NMR spectrum of the complex Pt(IV)-biSi-2, there is a signal at 7.80 ppm that integrates for four protons. This signal should correspond to the −NH_2_ groups of the isopropylamine moieties. To confirm this fact, a ^1^H–^195^Pt bidimensional HMBC experiment was performed (Figure S9). The resulting spectrum showed an intense signal at 1370 ppm (^195^Pt) /7.80 ppm (^1^H), which undoubtedly indicates a clear correlation between the −NH_2_ groups and the Pt center. In addition, a less intense signal appears in the bidimensional spectra at 1370 ppm of ^195^Pt/3.29 ppm of ^1^H, which corresponds to the correlation of the −CH groups of the ipa with the platinum atom. The analysis of the FTIR spectra (Figures S7 and S10) also unequivocally confirms the formation of the desired compounds. The above-mentioned band of the ν (−OH) observed for the Pt(IV) precursor has disappeared as a result of the formation of the carbamate group, which shows an intense band around 1650–1630 cm^–1^ corresponding to the O–C=O–N vibration. Moreover, a strong doublet around 1100–1050 cm^–1^ is observed in both spectra, which is due to the Si-(OCH_2_CH_3_) vibration.^36^ Finally, both Pt(IV) complexes were also characterized by mass spectrometry using electrospray ionization (positive mode), obtaining in each spectrum (Figure 2A,B) a molecular peak corresponding to the ionized complex. Moreover, the platinum isotopic distribution profile in each signal unambiguously matches with the theoretical simulation. The purity of these Pt(IV) complexes with bis-organosilane ligands was determined by HPLC (Figure S8 for Pt(IV)-biSi-1 and Figure S11 for Pt(IV)-biSi-2) and elemental analysis (see the Experimental Section), obtaining with both techniques a purity >95%.

To study the morphology of the final solids of both complexes (Pt(IV)-biSi-1 and Pt(IV)-biSi-2) and their composition and distribution of the different elements in each one, transmission electron microscopy STEM/EDS mapping analyses were performed.^37^ As can be seen from the electron images in Figure 2C,D, Pt(IV)-biSi-2 showed a denser structure, whereas Pt(IV)-biSi-1 presented a porous sponge-like morphology. Images of both complexes taken with a camera coupled to a DRX instrument confirm this fact (Figure S12A,B). This aspect was very relevant because the two complexes only differ in the amines directly bound to the metal center: two ammonia groups in the case of Pt(IV)-biSi-1 and two ipa moieties in the complex Pt(IV)-biSi-2. On the other hand, the EDS mapping technique showed that the three elements analyzed, i.e., platinum, chlorine, and silicon, are homogeneously distributed over the surface of both solids, confirming again the formation of the complexes and the suitability of the synthetic pathway chosen.

### Reduction Assays

Pt(IV) complexes used in cancer chemotherapy are considered to be prodrugs that need to be reduced to the Pt(II) active species by different biomolecules inside the cell to exert biological activity. This is because they are very stable and rather chemically inert, with slow ligand exchange rates.^6,11^

To determine the reducing capacity of Pt(IV)-biSi-1 and Pt(IV)-biSi-2 in reactions against biomolecules and the Pt(II) species generated in the media, we have evaluated their reactivity toward ascorbic acid (AsA) and glutathione (GSH) (Scheme S1), which are the main reducing agents present in the human body.^11^ AsA presents an intracellular concentration of 1 mM, whereas the GSH concentration is 2 mM.^38^ This reactivity was monitored by ^195^Pt NMR at different times. Thus, by analyzing the different regions where the signals of Pt(II)/Pt(IV) species appear, it is possible to identify and assign the different compounds formed during the reaction. In most cases, the regions where the Pt(II) and Pt(IV) signals appear are separated by more than 3000 ppm.^33^

We first studied the reduction of the complexes against AsA, monitoring the reaction in both the Pt(IV) and Pt(II) region at 2, 6, and 24 h. In both cases, we used a mixture of DMSO/D_2_O (4:1) to reach the solubilization of the complexes and the AsA. In the Pt(IV)-biSi-1 reduction experiment, during the first hour, only the signal at 1210 ppm appeared in the Pt(IV) region (Figure S13A), which corresponds to the original complex, and no signal was detected in the Pt(II) region. However, after 24 h, this signal underwent a marked decrease in intensity, with a new signal appearing in the Pt(II) region at −2110 ppm (Figure 2E), which corresponds unequivocally to cisplatin, confirming that the reduction process took place. This fact is in agreement with the reduction of its Pt(IV) precursor, oxoplatin, which is also reduced to cisplatin after 24 h of reaction with AsA.^9,39^

In contrast, the Pt(IV)-biSi-2 compound showed a much faster reduction rate with AsA, as no signals were observed in the Pt(IV) region after reaction for 2 h (Figure S13B), whereas the signal of its Pt(II) homologue, *cis*-[PtCl_2_(ipa)_2_], appeared in the Pt(II) region (Figure 2F). This is also in agreement with previous studies on the reduction of iproplatin with AsA.^39,40^ Furthermore, this signal decreased with time, with another signal appearing after 6 h at 2010 ppm, a typical value for the PtN_2_ClO fraction.^33,41^ This fraction was assigned to the formation of the monoaquo species, *cis*-[PtCl(ipa)_2_(OH_2_)]^+^, due to substitution of a chloride ligand by a water molecule. After 24 h, the intensity of this signal increased markedly and became the dominant species in the medium. This rapid reduction of the Pt(IV)-biSi-2 compound compared to the other bis-organosilane complex may be related to the high cytotoxic activity of this drug on tumor cells, as will be discussed below. In fact, the monoaquo species derived from cisplatin is more reactive than the diaquo form and shows high activity against biological targets.^3^

The reactivity of both complexes toward the reducing agent GSH was also studied using the DMSO/D_2_O (4:1) mixture. It can be seen how the signal corresponding to the Pt(IV)-biSi-1 compound decreases with time, although at 24 h, there is still a fraction of free complex without undergoing reduction (Figure S13C). In the Pt(II) region, two signals appeared after 24 h at −2728 and −3405 ppm (Figure 2G). The first value is consistent with the *cis-*PtN_2_SX fraction,^41,42^ meaning that this species, once reduced to its Pt(II) homologue, coordinates with GSH by displacing a chloride ligand. The second signal corresponds to another Pt(II) moiety with more than one ligand containing sulfur atoms. It is important to note that in *cis* Pt(II) compounds, when a ligand with a sulfur-donor group displaces the first chloride, the ligand is left in trans position to an amine species, which is now susceptible to substitution by another ligand due to the strong trans effect exerted by the GSH molecule.^43^ Thus, the presence of several ligands with sulfur atoms in the Pt(II) complex results in a large signal shift at high δ values.^42^ On the other hand, the reduction pathway of the Pt(IV)-biSi-2 complex gives two signals with similar values in the Pt(II) region (Figure 2H), which could indicate a similar reduction mechanism of both Pt(IV) complexes with bis-organosilane ligands against GSH. However, for the Pt(IV)-biSi-2 complex, the signal corresponding to the Pt(IV) complex practically disappeared after 24 h (Figure S13D), as was also the case for AsA. Therefore, the Pt(IV)-biSi-2 complex seems to be more sensitive to the reduction process by these two biomolecules than Pt(IV)-biSi-1. Moreover, in both cases, the reduction process with GSH rendered different Pt(II) species than those obtained with oxoplatin and iproplatin.^9,44^ This in turn could be directly related to the higher cytotoxic activity of these complexes compared with cisplatin, as will be discussed below.

### Biological Impact of Pt(IV) Compounds on Human Intestinal Cells

The response of intestinal tumor (HCT 116 and HT-29) and nontumorigenic intestinal cells (HIEC6) to cisplatin treatment was determined using MTT (3-[4,5-dimethylthiazole-2-yl]-2,5-diphenyltetrazolium bromide) assays that estimate cell viability, as indicated in the methods section. The half maximal inhibitory concentration or inhibitory concentration 50 (IC_50_) values were determined after 48 h of treatment (Figures S14A,B). HCT 116 cells were more resistant, as previously reported.^45^ Consistent with this, dose–response experiments revealed that nontumorigenic intestinal cells (HIEC6) were slightly more sensitive to cisplatin than HCT 116 tumor cells (not shown). However, tumor (HCT 116) and nontumorigenic intestinal (HIEC6) cells exhibited very close IC_50_ for cisplatin, and this lack of discrimination unveils the necessity to improve the profile. This could be achieved by reduction of doses through extended treatment duration, combining cisplatin with other treatments, or modifying platinum compounds to improve selectivity between tumoral and healthy cells in the colon. We explored the latest cisplatin modifications that improve the response of tumor cells to platinum compounds without affecting healthy cells.

**Figure 3:**
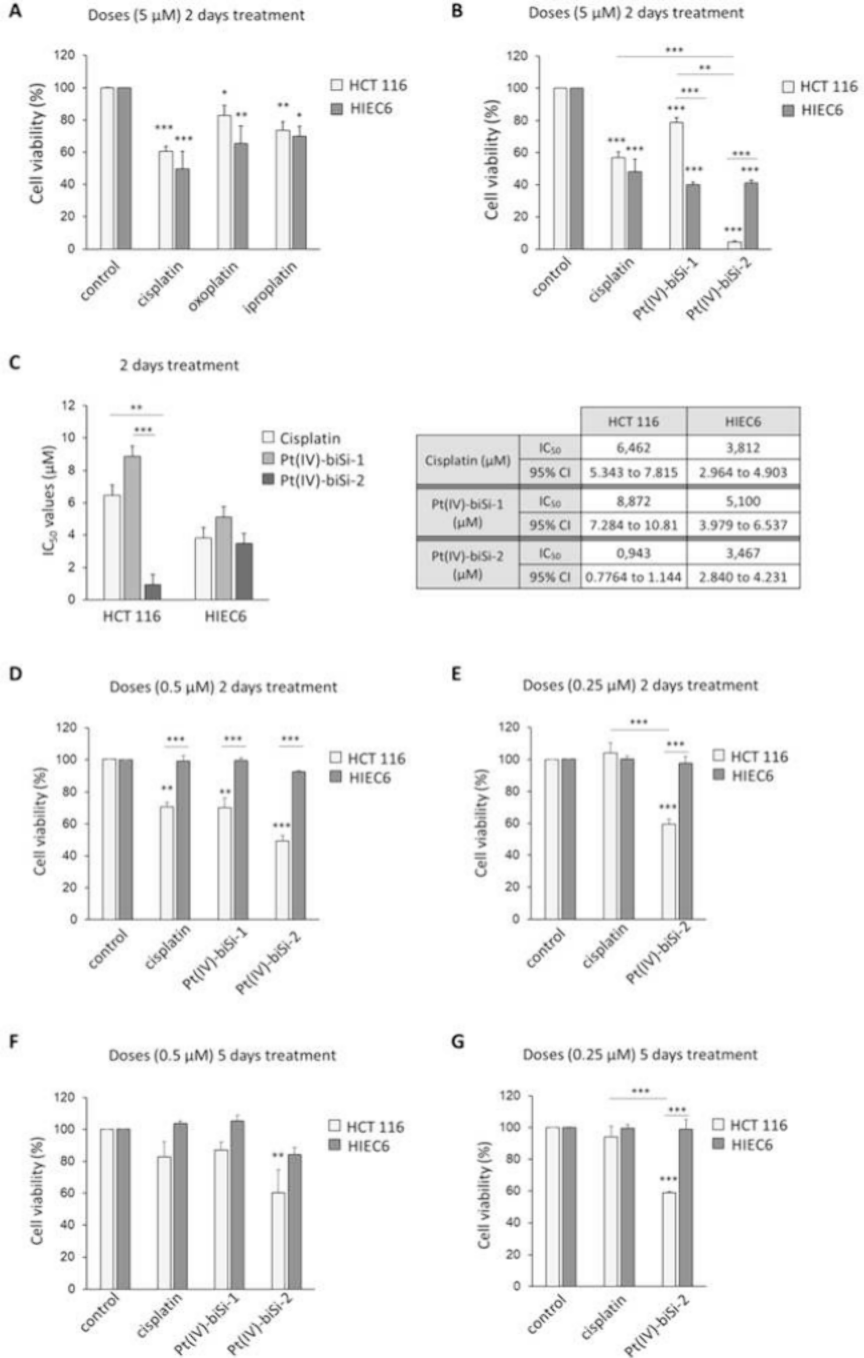
Biological impact of platinum compounds on human intestinal cancer and healthy cells. MTT assays (A, B, and D–G) to measure viability of HCT 116 (cancer) and HIEC6 (healthy) intestinal cells in response to treatment with indicated compounds and (C) determination of half maximal inhibitory concentration (IC50). (A) Forty-eight hour treatments with 5 μM platinum intermediates oxoplatin and iproplatin and (B) Forty-eight hour treatments with 5 μM Pt(IV)-biSi-1 and Pt(IV)-biSi-2 compared with cisplatin. (C) IC_50_ values for cisplatin, Pt(IV)-biSi-1, and Pt(IV)-biSi-2 on tumor (HCT 116) and healthy (HIEC6) human intestinal cell lines. (D) Forty-eight hour treatments with 0.5 μM Pt(IV)-biSi-1 and Pt(IV)-biSi-2 and (E) Forty-eight hour treatments with 0,25 μM Pt(IV)-biSi-2. (F) Five days of treatment with 0,5 μM cisplatin, Pt(IV)-biSi-1, or Pt(IV)-biSi-2 and (G) Five days of treatment with 0,25 μM cisplatin or Pt(IV)-biSi-2. Cell viability is expressed as a percentage. Values are the mean ± SEM of three independent experiments. In all cases, the results are compared to the ones obtained with cisplatin at the same doses.

The biological activity of intermediates (oxoplatin and iproplatin) and final Pt(IV) complexes (Pt(IV)-biSi-1 and Pt(IV)-biSi-2) was evaluated in tumor (HCT 116) and nontumorigenic (HIEC6) intestinal cell lines. Based on previous works, an initial concentration of 5 μM was chosen to determine the IC_50_ for cisplatin in our cell models (Figure S14A). Pt(IV) intermediates displayed an overall weaker cytotoxicity than cisplatin with 20 to 30% reduction in the viability of tumoral and healthy cells, respectively, after 2 days of treatment (Figure 3A). Thus, both oxoplatin and iproplatin were more toxic to healthy cells than to cancer cells (Figure 3A). In contrast, Pt(IV)-biSi-2 exhibited maximal cytotoxicity for cancer cells and reduced their viability by 90%, showing a potency remarkably greater than that of cisplatin (Figure 3B). In contrast, Pt(IV)-biSi-1 caused a milder reduction in cancer cell viability (20%) while remaining as toxic to healthy cells as cisplatin or Pt(IV)-biSi-2 (60% reduction in cell viability), although cisplatin, Pt(IV) intermediates, and final complexes reduced the viability of both cancer (HCT 116) and healthy cells (HIEC6). Interestingly, Pt(IV)-biSi-2 was extremely toxic to cancer cells and much less to healthy cells, a selectivity that could be exploited.

Because Pt(IV)-biSi-2 appeared to kill most tumor cells at the concentrations normally used for cisplatin but was still cytotoxic for healthy cells, we wondered if a dose reduction would allow us to exploit the differential sensitivity of tumor and nontumorigenic intestinal cells. Thus, we compared the IC_50_ of cisplatin, Pt(IV)-biSi-1, and Pt(IV)-biSi-2 on these cell lines (Figure 3C). Cisplatin and Pt(IV)-biSi-1 show small differences between healthy (HIEC6) and tumoral (HCT 116) cells, and their IC_50_ was similar for the same cell lines. In contrast, cancer cells were strongly responsive to Pt(IV)-biSi-2 with an IC_50_ largely reduced compared to their IC_50_ for Pt(IV)-biSi-1 or cisplatin. Notably, HIEC6 nontumorigenic intestinal cells were less sensitive (bigger IC_50_) to Pt(IV)-biSi-2 than HCT 116 tumor cells (black columns on Figure 3C left). The IC_50_ of Pt(IV)-biSi-2 for tumor cells was 0.94 μM, whereas the IC_50_ for healthy cells was 3.47 μM (Figure 3C right); that is, a 3.7-fold higher concentration was needed to reduce nontumorigenic intestinal cell viability by 50%. Indeed, GSH is overexpressed in cancer cells,^46,47^ which could explain the increased toxicity for cancer versus healthy intestinal cells. Detailed dose–response viability curves for healthy and tumor intestinal cells versus Pt(IV)-biSi-1 and Pt(IV)-biSi-2 are presented (Figure S14C,D).

Based on this, we selected a 1/10th lower concentration, 0.5 μM Pt(IV)-biSi-1 and Pt(IV)-biSi-2, to analyze the response of nontumorigenic intestinal or tumor cells to acute treatment (2 days). Cisplatin (0.5 μM) still induced ∼30% cytotoxicity in tumor cells (Figure 3D) and Pt(IV)-biSi-1 and Pt(IV)-biSi-2, also significantly reducing the viability of tumor cells at this dose, with the effects of Pt(IV)-biSi-1 being very similar to those of cisplatin. Remarkably, 0.5 μM Pt(IV)-biSi-2 reduced the viability of HCT 116 tumor cells by 50% (Figure 3D), and a further dose reduction by half (0.25 μM) enhanced the differential effects of Pt(IV)-biSi-2 which reduced cancer cell viability by 40% without affecting nontumorigenic intestinal cells (Figure 3E). Notably, cisplatin and the Pt(IV) complexes rendered similar results in other alternative colon cancer cell lines, such as HT-29, (Figure S14E), further indicating a strong cytotoxic effect of Pt(IV)-biSi-2 on colon cancer cells. An approach to chronic treatment conditions was made by extending the treatment to 5 days. Chronic treatment of HCT 116 cancer cells with 5 μM Pt(IV)-biSi-2 generated about 8–10 fold greater cytotoxicity than cisplatin since only 5% cells survived, whereas more than 50% of the cells survived to cisplatin (Figure S14F). However, Pt(IV)-biSi-1 did not improve its selectivity or efficacy over cisplatin, which may reflect depletion or instability of these drugs over long periods of time. Importantly, 5 days of treatment with this 1/10 dose reduction (0.5 μM) of Pt(IV)-biSi-2 reduced tumor cell viability by 40%, which is comparable to the 50% reduction caused by the same doses in 2 days, suggesting better stability of this compound (Figure 3F). Interestingly, a further dose reduction (0.25 μM) in chronic treatment (5 days) enhanced the differential effects of Pt(IV)-biSi-2 (Figure 3G) and improved its profile versus cisplatin. Chronic treatment with 0.25 μM of cisplatin had no effect on any cells, whereas Pt(IV)-biSi-2 caused a 40% reduction of tumor cell viability without affecting nontumorigenic intestinal cells (Figure 3G).

The cytotoxicity of the compounds was estimated using an alternative method to confirm previous results. Viability was estimated by trypan blue staining, a dye that is actively excluded only by viable cells. Treatment with cisplatin or Pt(IV)-bi-Si-1 (0.5 μM) for 2 days reduced viability by 20%, while Pt(IV)-bi-Si-2 reduced viability by approximately 60% (Figure S15A,B). Treatment for 2 days with 0.25 μM Pt(IV)-biSi-2 still reduced by 40% the viability of colon cancer cells with very little effect of cisplatin (Figure S15C,D). Moreover, using flow cytometry as an additional alternative estimation of the cytotoxicity of these compounds confirmed a stronger effect of Pt(IV)-biSi-2 on (HCT 116) cancer cells and a similar insensitivity on intestinal noncancer cells (HIEC6) (data not shown).

Taken together, the results indicated that Pt(IV)-biSi-2 is more cytotoxic for cancer cells than cisplatin and Pt(IV)-biSi-1 and maintained its cytotoxicity at lower doses and for longer periods (5 days). Interestingly, although both Pt(IV) final derivatives exhibited similar cytotoxicity to healthy intestinal cells, Pt(IV)-biSi-1 was more cytotoxic to healthy than to tumoral intestinal cells, whereas Pt(IV)-biSi-2 was more cytotoxic to tumor cells than to healthy intestinal cells.

In this work, we have synthesized and evaluated the cytotoxicity of a new Pt(IV) based complex, Pt(IV)-biSi-2, with differential cytotoxicity on human intestinal cancer or nontumorigenic intestinal cells (HCT 116 and HIEC6). We demonstrate a selective cytotoxic effect of Pt(IV)-biSi-2 at a very low (0.25 μM) concentration, lower than standard cisplatin doses^48^ and at which no cytotoxicity was detected on healthy intestinal HIEC6 cells. Pt(IV)-biSi-2 at 0.25 μM reduced the number of living cells by approximately 40% after 48 h of treatment. The effective Pt(IV)-biSi-2 concentration was at least 5 times lower than that reported for cisplatin (5–25 μM) to induce CRC cell cycle arrest, alter mitochondrial respiration,^49^ and cause apoptosis.^50−52^ Thus, this prodrug is more potent and displays long-lasting action on the final cell fate balance, as shown in chronic treatments. The effects of 0.25 μM Pt(IV)-biSi-2 at 48 h suggest fewer adverse side effects on healthy cells and a different mechanism of action from cisplatin.

### Lipophilicity of Pt(IV) Complexes

The differences in biological activity exhibited by Pt(IV) complexes *in vitro* in both tumor and healthy cells may be related to their lipophilic behavior. We therefore decided to evaluate their lipophilicity by theoretically determining the partition coefficient (logP), the ratio of the concentrations of the compounds in two immiscible solvent phases (noctanol and water) at equilibrium. The latter parameter indicates the degree to which a molecule is hydrophilic or lipophilic. The theoretical simulation gave logP values of 3.465 for Pt(IV)-biSi-1 and 5.469 for Pt(IV)-biSi-2. Both values of logP are far from the value of −2.4 reported for cisplatin.^53^ These results showed the higher lipophilicity of Pt(IV)-biSi-2, confirming that the incorporation of two isopropylamine molecules with respect to ammonia in the equatorial positions leads to a significant increase in its lipophilicity.

### Stability of Pt(IV) Complexes

**Figure 4:**
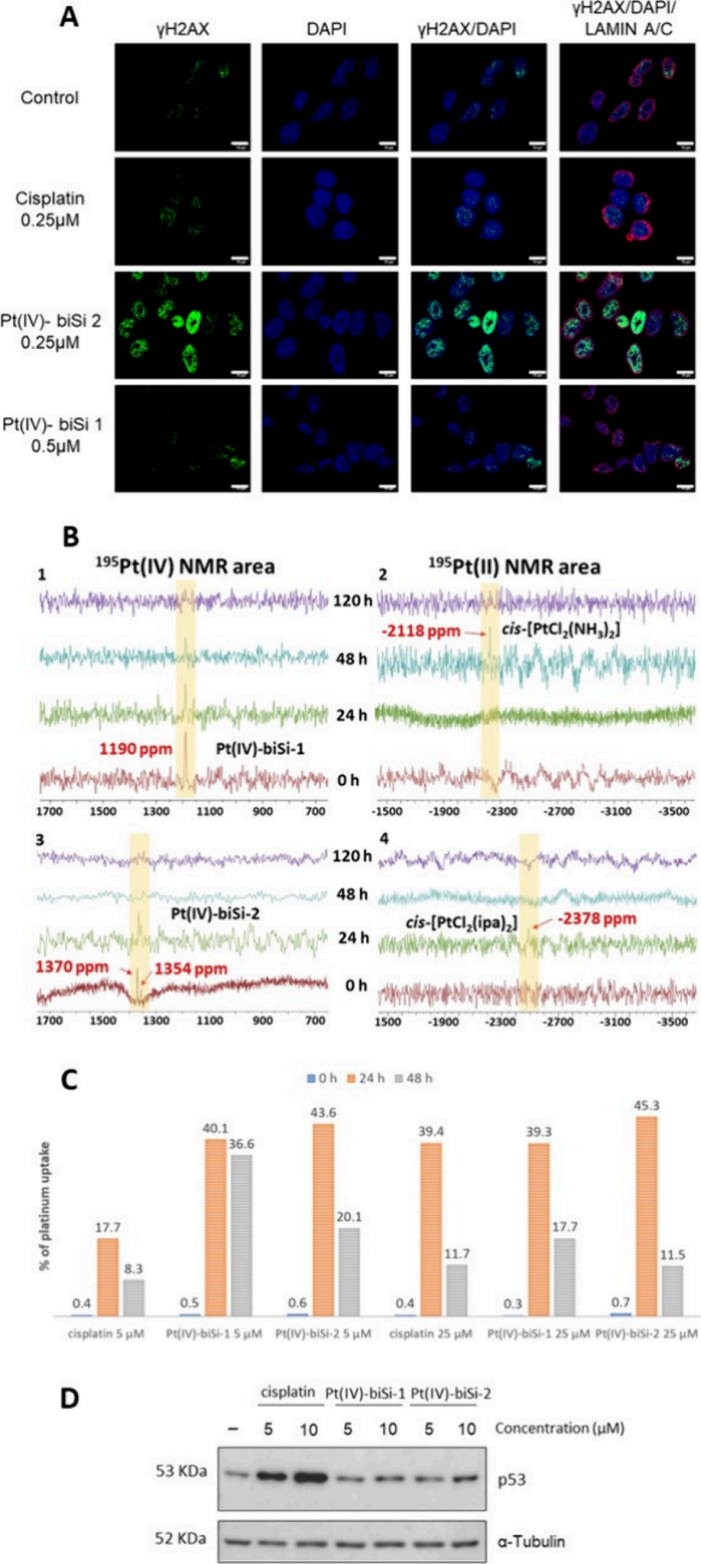
(A) Confocal imaging of DNA damage foci induced by treatment with the indicated compounds for 48 h; histone gH2AX (green) marks unresolved double-strand DNA breaks; DAPI (4,6-diamidino-2-phenylindole dihydrochloride) stains the DNA (blue); Lamin A/C marks the nuclear envelope (red) of HCT 116 colon cancer cells; doses as indicated; magnification bar: 10 μm. (B) Time course of ^195^Pt NMR spectra of Pt(IV)-biSi-1 (upper panels 1 and 2) and Pt(IV)-biSi-2 (lower panels 3 and 4) in DMEM; Pt(IV) and Pt(II) regions are highlighted with yellow shadows; different colors are assigned to different times. (C) ICP-OES measurement of the % of platinum uptake by HCT 116 colon cancer cells exposed to 5 μM of cisplatin, Pt(IV)-biSi-1, and Pt(IV)-biSi-2 for different times (as indicated). (D) Western blot analysis of p53 protein levels in HCT 116 cells treated with cisplatin, Pt(IV)-biSi-1, and Pt(IV)-biSi-2 for 48 h at doses indicated. Tubulin as a loading control showed similar loading in all lanes; differences in p53 intensity reflect p53 induction.

We then decided to evaluate the stability of the two Pt(IV) complexes in the medium (DMEM) used to carry out the *in vitro* experiments, monitoring their kinetics by ^195^Pt NMR at different times to evaluate the possible species formed over time. DMEM is supplemented with proteins and other molecules essential for cell growth (Experimental Section). Thus, similar to the reduction assays, we analyzed the Pt(II)/Pt(IV) region where the expected species appear. As shown in Figure 4B1, the signal corresponding to the compound Pt(IV)-biSi-1 in the Pt(IV) region at 1190 ppm decreases with time up to 48 h, where a small fraction of the initial complex remains intact. At this time, the signal clearly corresponding to cisplatin had appeared in the Pt(II) region (Figure 4B2) as a result of the reduction of the original compound in the media. However, after 5 days, no species appeared in either region of these spectra because of the complete disappearance of the Pt(IV) prodrug and the binding of the Pt(II) species derived from cisplatin to the proteins present in the media, which formed a precipitate after this time. On the other hand, the signals corresponding to the two isomers of the Pt(IV)-biSi-2 complex in the Pt(IV) region (Figure 4B3) suffered a marked decrease after 24 h, having appeared at this time a signal in the Pt(II) region at −2371 ppm (Figure 4B4) corresponding to its Pt(II) counterpart, the compound *cis*-[Pt(ipa)_2_Cl_2_]. Both Pt(IV)/Pt(II) signals disappeared after 48 h, most likely again because of the binding of the complex to the proteins. Thus, the Pt(IV)-biSi-2 complex is less stable in this medium and also shows a faster rate of reduction than the other compounds, in agreement with the results obtained in the reduction assays.

### Platinum Internalization in the HCT 116 Cells

To determine and quantify the amount of platinum that the tumor cells are able to uptake during their exposure to the metallodrugs, we performed a new treatment of these cells with both Pt(IV) bis-organosilane complexes, using cisplatin as reference, at two different concentrations (5 and 25 μM) at different times and measured the platinum concentrations in the cells and in the biological media by inductively coupled plasma optical emission spectroscopy (ICP-OES). Cells were treated with these platinum complexes at the two concentrations and collected at times 0, 12, 24, and 48 h. Treated cells were pelleted by centrifugation, and the supernatant was kept to measure the noninternalized platinum. The cell pellet was digested in a solution of concentrated HNO_3_ to release the internalized platinum. Measurements of the platinum from cells and free platinum in the media were performed by ICP-OES (Figure 4C). The amount of platinum internalized increased in all the cases up to 24 h, but after 48 h, the concentration of platinum in the cell pellet significantly decreased, likely because of the death of most of the cells, causing a large amount of this metal to be released into the media (negligible values of less than 1% for platinum uptake at 0 h are due to experimental errors in the separation of the cell pellet from the supernatant). The amount of platinum internalized by the cells treated with 5 μM of the two Pt(IV) prodrugs at short times is higher than that of cisplatin. This fact may be related to the higher lipophilicity of the two bis-organosilane Pt complexes compared to cisplatin, as discussed in the Lipophilicity of Pt (IV) Complexes section. Treatment of cells with 25 μM of the prodrugs caused high and rapid cell mortality, making it difficult to observe differences.

### Pt(IV) Bis-organosilane Complexes Reduce Cancer Cell Viability through Mechanisms Distinct from Cisplatin

Cisplatin is known to induce DNA damage, which increases levels and activity of the tumor suppressor protein p53.^54^ p53 stimulated by DNA lesions produces cell cycle arrest and activates apoptotic mechanisms if they are not properly repaired.^54^ Fifty percent of human solid tumors bear p53 mutations, and the sensitivity to cisplatin is attributed to the presence of wild-type p53.^55^ HCT 116 cancer cells were exposed to increasing concentrations of cisplatin or Pt(IV)-biSi-1 or -2 for 48 h, and the effects on DNA damage and p53 levels were analyzed. Confocal imaging of the DNA damage response was followed by immunofluorescence of histone gH2Ax foci at double-strand DNA breaks (DSB) (Figure 4A). Consistent with previous results, unresolved DSB or histone H2Ax foci were retained at 48 h in cells treated with 0.5 μM Pt(IV)-biSi-2 but not with similar doses of cisplatin or Pt(IV)-biSi-1. Thus, the inability to properly repair DSB and maintain genomic stability distinguishes Pt(IV)-biSi-2 from other platinum compounds. Moreover, Western blotting of total cell lysates (Figure 4D) showed that cisplatin increased p53 levels (at all concentrations), as previously reported.^56^ However, none of the Pt(IV) prodrugs were able to increase p53 levels even at concentrations higher than those needed to reduce cell viability. This, together with the differential effects on cancer and healthy cells, suggests a different mechanism of cancer cell death between cisplatin and Pt(IV) bis-organosilane complexes, possibly involving p53-independent death pathways.

### *In Vivo* Significance

**Figure 5:**
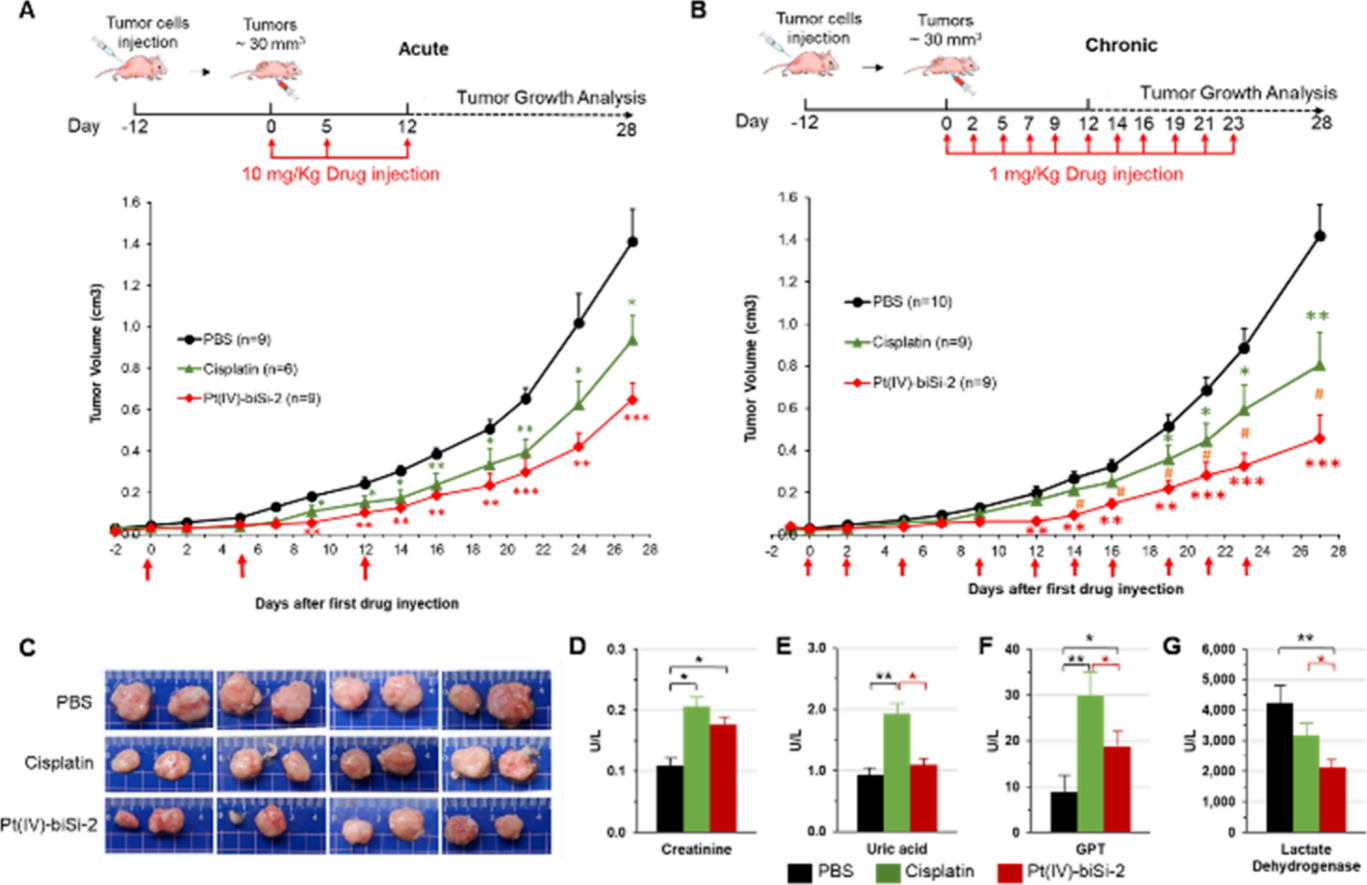
Pt(IV)-biSi-2 antitumoral efficacy in *in vivo* treatment. Tumor growth evaluation after treatment with PBS (black circles), 4170 or 417 μM cisplatin (green triangles), and 1370 or 137 μM Pt(IV)-biSi-2 (red diamonds) in acute (A) and chronic (B) treatment, respectively, in intraperitoneal injections (indicated with red arrows). The schematic of the experiment is shown at the top of the panels A and B. Numbers of tumors analyzed in each group are indicated in the legend. Statistical analysis was performed with the *t* test. *, ^#^*p* < 0.05; **p < 0.01; ***p < 0.001. Asterisks (*) with the color of the treatment represent significance with respect to controls (PBS); pads (#) represent significance comparing both treatments. (C) Representative tumors in chronic treatment in each condition are shown. (D) Renal toxicity estimated from plasma levels of creatinine; hepatic toxicity estimated from plasma levels of uric acid (E) and GPT (F) in animals after each treatment. (G) Lactate dehydrogenase (LDH) levels are a marker of tumor activity. The average values of creatinine, uric acid, GPT, and LDH obtained in the serum of the animals of each group are shown. Statistical analysis was performed with the *t* test. *p < 0.05; **p < 0.01; ***p < 0.001.

The *in vitro* results encouraged us to explore the translational potential in a preclinical *in vivo* model of nude mice with grafted tumor cells. The effects of Pt(IV)-biSi-2 were compared to the FDA-approved drug cisplatin in acute and chronic treatment. Tumors were generated by subcutaneous injection of human colon cancer cells (HCT 116), and when tumors reached around 0.03 cm^3^, three mice groups were established: group 1: control mice nontreated/PBS; group 2: mice treated with cisplatin (1 mg/kg or 417 μM for acute or 10 mg/kg total or 4170 μM for chronic treatment); and group 3: mice treated with Pt(IV)-biSi-2 (1 mg/kg or 137 μM for acute and 10 mg/kg or 1370 μM for chronic treatment). Tumor growth was monitored until tumors in the control group reached the ethically allowed size. Both cisplatin and Pt(IV)-biSi-2 significantly reduced tumor volume in both acute (Figure 5A) and chronic (Figure 5B) treatments. Importantly, Pt(IV)-biSi-2 reduced tumor growth more than cisplatin in both acute and chronic treatments, and the differences were statistically significant, even though Pt concentrations in Pt(IV)-biSi-2 were one-third that of cisplatin (Figure 5A,B). Tumors derived from mice treated were smaller than those from controls, and those from mice treated with Pt(IV)-biSi-2 were smaller than tumors from mice treated with cisplatin (Figure 5C). Notably, both renal toxicity (estimated by measuring creatinine) and hepatic toxicity (estimated from uric acid and GPT or glutamic pyruvic transaminase) levels in plasma were significantly lower with Pt(IV)-biSi-2 than with cisplatin (Figure 5D–F). Contrary to what we observed with cisplatin, tolerance to Pt(IV)-biSi-2 treatment was excellent, both acutely and chronically, and at drug concentrations that are common and very high for these treatments (Figure S16). No side effects (weight loss greater than 5% or abnormal postures) or deaths of the mice were observed during treatment with Pt(IV)-biSi-2, in contrast to what occurred with cisplatin. On the other hand, many types of cancer cause a general increase in lactate dehydrogenase (LDH), and it is considered a nonspecific tumor marker.^57^ Both treatments reduced LDH levels compared to those of untreated animals (PBS), reflecting the response to treatment. However, Pt(IV)-biSi-2 treatment reduced LDH levels significantly more (Figure 5G), which confirms the superior efficiency of Pt(IV)-biSi-2 to halt tumor growth. Taken together, the *in vitro* and *in vivo* results indicate that Pt(IV)-biSi-2 is stable in biological fluids, has higher antitumor efficacy and less off-target toxicity than cisplatin, and allows chronic treatments that are better tolerated.

The biological activity of the prodrug Pt(IV)-biSi-2 *in vitro* and *in vivo* and in particular its *in vitro* selectivity for cancer cells and coherently milder *in vivo* toxicity indicate that it is a strong candidate to replace cisplatin and analogues. As a prodrug, Pt(IV)-biSi-2 could be used to design chemotherapeutic nanoparticles that overcome the limitations of classical Pt-based chemotherapy. Inorganic chemistry has been indisputably valuable for chemotherapy, but its limitations should be overcome, and the irruption of nanotechnology could be strategic to resolve these drawbacks. For example, poor pharmacokinetic profiles, low specificity, rapid metabolism and excretion, recognition, and elimination by the immune system are barriers that can be avoided using nanoparticles. Intense research with nanoplatforms including organic nanoformulations (liposomes, polymeric micelles, hydrogels, polymeric nanospheres, and nanocapsules) or inorganic ones (carbon nanotubes, gold, silica, iron oxide, etc.) will lead to alleviating the side effects associated with platinum-based chemotherapy in cancer patients.^58,59^ In particular, mesoporous silica nanoparticles (MSNs)^60−69^ are excellent candidates to incorporate bis-organosilane Pt(IV) complexes because of their excellent physicochemical properties and the clear chemistry intersection between both Si moieties. Thus, grafting, adsorption, direct synthesis, etc., could be used to couple Pt(IV)-biSi-2 into nanoparticles that may overcome current chemotherapeutic limitations. Future research in our group will explore the potential of nanoparticles using this prodrug.

## Conclusions

Pt(IV) complexes provide a new approach to Pt(IV) prodrug design, allowing the incorporation of different ligands in different positions while retaining the pharmacological activity of Pt(II). Thus, we have synthesized two Pt(IV) prodrugs with bis-organosilane ligands in the axial positions from their Pt(IV) precursors (oxoplatin and iproplatin) with the aim of improving the cytotoxic activity and selectivity of these compounds against tumoral cells. The reaction of the Pt(IV) precursors with the isocyanate group of the silane moieties directly yields the desired complexes via the formation of a carbamate group. ^195^Pt NMR assays after exposure to reducing agents, AsA and GSH, indicated that both complexes are reduced to different Pt(II) species from their Pt(IV) precursors, oxoplatin and iproplatin Moreover, Pt(IV)-biSi-2 shows a faster reduction rate than Pt(IV)-biSi-1. This may explain the improved *in vitro* efficiency (increased antiproliferative activity) of the Pt(IV)-biSi-2 complex against colorectal tumor cell lines. In addition, Pt(IV)-biSi-2 showed less cytotoxicity against control, nontumorigenic intestinal cells, that is, enhanced selectivity toward tumor cells. Moreover, ICP measurements showed that the Pt(IV) complexes were more effectively internalized by cells than cisplatin. In addition, induction of the p53 pathway by cisplatin but not by Pt(IV) compounds highlights different cancer cell death mechanisms. Our results support at first the use of this prodrug as a potential alternative to the use of cisplatin and its analogues. The advantages of Pt(IV)-biSi-2 are a reduced IC_50_ in human colorectal cancer cells, which is similar for HCT 116 and HT-29 and already four times lower than that for standard *in vitro* treatment. This property allows the identification of a suitable selectivity window, i.e., a sufficiently low dose of Pt(IV)-biSi-2 to be cytotoxic for tumor but not to healthy intestinal cells. Finally, *in vivo* experiments in a preclinical mouse model harboring engrafted colorectal tumors revealed that Pt(IV)-biSi-2 achieved greater inhibition of tumor growth at lower concentrations than cisplatin and with fewer toxic side effects. In summary, Pt(IV)-biSi-2, as a prodrug, addresses the limitations associated with classical Pt-based chemotherapy by offering improved selectivity, reduced side effects, and enhanced stability. We are currently exploring its nanotechnological potential by including this prodrug in nanoparticles designed to combine treatments that work synergistically to further advance cancer treatment.

## Experimental Section

### Materials and Methods

#### Chemicals

Cisplatin (99% Pt) and potassium tetrachloroplatinate (II) (99.9% Pt) were purchased from Strem Chemicals Inc. (Newburyport, MA, USA). Thiazolyl blue tetrazolium bromide (MTT), hydrogen peroxide (30% w/v), isopropylamine (>99.5%), 3-(triethoxysilyl)propyl isocyanate (95%), chloroform, ethanol, hexane, petroleum ether, *N*,*N*-dimethylformamide (anhydrous, 99.8%), dimethyl sulfoxide, and the deuterated solvents dimethyl sulfoxide-*d*_6_ (99.8% D) and deuterium oxide (99.9% D) were purchased from Sigma-Aldrich (Madrid, Spain). DMEM and Opti-MEM media were purchased from Lonza (Basel, Switzerland).

#### Characterization Techniques of Pt Complexes

^1^H and ^13^C NMR measurements were performed on a Varian Infinity 400 MHz spectrometer fitted with a 9.4 T magnetic field (URJC, Móstoles, Spain) at room temperature (25 °C). Chemical shifts (δ) are shown in ppm, and they were externally referenced to tetramethylsilane (TMS). The kinetics of reduction of the complexes against reducing agents was monitored by ^195^Pt NMR at 37 °C on a Bruker Avance II/500 MHz spectrometer fitted with a 11.75 T magnetic field (URJC, Móstoles, Spain). Chemical shifts (δ) are shown in ppm, and they were externally referenced to potassium hexachloroplatinate (IV) (K_2_PtCl_6_) in D_2_O (0 ppm). Mass measurements were performed on an ultrahigh-performance liquid chromatography-tandem mass spectrometry (UHPLC-HESI-MS/MS) using a VIP heated electrospray ionization interface (Bruker UHPLC/MSMS EVOQ ELITE) with a triple-quadrupole detector (URJC, Móstoles, Spain). FTIR analyses were collected, using the KBr buffer technique, on a Mattson Infinity series apparatus in the wavelength range from 4000 to 400 cm^–1^ with a step size of 2 cm^–1^, and 64 scans were collected for each analysis (URJC, Móstoles, Spain). The elemental composition was studied using a CHNS-O analyzer Flash 2000 Thermo Scientific apparatus (URJC, Móstoles, Spain). The purity of both P(IV) complexes with bis-organosilane ligands by HPLC was determined by using a ultrahigh-performance liquid chromatography-tandem mass spectrometry (UHPLC-HESI-MS/MS) using the VIP heated electrospray ionization interface (Bruker UHPLC/MSMS EVOQ ELITE) with a triple-quadrupole detector (URJC, Móstoles, Spain). Scanning transmission electron microscopy (STEM) and energy dispersive X-ray spectrometry (EDS) image analyses were performed on a JEOL JEM 2100 electronic microscope operating at 200 kV equipped with a CCD ORIUS SC1000 (Model 832) camera (Complutense University of Madrid, Madrid, Spain). Images of the solids were captured with a camera coupled to an X-ray diffractometer D8 Venture (Bruker) (URJC, Móstoles, Spain). The ICP-OES analysis was performed in an ICP-OES Agilent 5800 VDV mass spectrometer using axial configuration and an aqueous matrix (URJC, Móstoles, Spain). The calibration of the device used was from 189 to 789 nm, and the resolution was 1 pm.

### Synthesis of the Pt Complexes

#### *cis-*Dichloro(diisopropylamine)platinum(II)

This complex was synthesized following the protocol published previously^70^: isopropylamine (395 μL, 4.8 mmol, 4 equiv) was added over a solution of 500 mg of K_2_PtCl_4_ (500 mg, 1.20 mmol, 1 equiv) in 1 mL of H_2_O. The reaction was stirred for 24 h at room temperature in the dark. The pale-yellow solid was filtered off and washed with a cold mixture of water/ethanol (70/30), chloroform, and hexane, and the final residue was air-dried. Yield: 65%. ^1^H RMN (DMSO-*d*_6_, 400 MHz): δ 4.76 (s, 2H), 3.11 (sept, 1H, *J* = 6.3 Hz), 1.21 (d, 6H, *J* = 6.5 Hz). ^13^C RMN (DMSO-*d*_6_, 400 MHz): δ 47.8, 23.5. ^195^Pt RMN (DMSO-*d*_6_, 500 MHz): δ −2228. Anal. Calcd for C_6_H_18_Cl_2_N_2_Pt: C, 18.76%; H, 4.72%; N, 7.29%. Found C_6_H_18_Cl_2_N_2_Pt: C, 18.45%; H, 4.53%; N, 7.01%.

#### *cis*-Dichloro(diamine)-*trans*-dihydroxoplatinum(IV) (Oxoplatin)

This complex was synthesized as it is described in the bibliography^28,71^ but with some modifications: cisplatin (500 mg, 1.66 mmol) was dissolved in 12 mL of H_2_O at 70 °C in the dark. Then, 30 mL of aqueous hydrogen peroxide (30% w/v) was added dropwise, and the mixture was stirred at the same temperature for 24 h. After that, the light-yellow solution was cooled to room temperature and exposed for several hours to light to eliminate the excess hydrogen peroxide. Then, the volume was reduced *in vacuo* to induce the precipitation of yellow crystals, which were then washed with a minimal amount of cold water to eliminate the perhydrate form, stirring vigorously. Finally, the solid was filtrated and air-dried. Yield: 52%. ^195^Pt RMN (DMSO-*d*_6_, 500 MHz): δ 854. IR (KBr, cm^–1^): 3520 ν(−OH); 3260 ν(−NH); 1590 δ(−NH); 1040 δ(Pt–OH); 557 ν(Pt–N); 455 ν (Pt–O). Anal. Calcd for H_8_Cl_2_N_2_O_2_Pt: H, 2.41%; N, 8.39%. Found H_8_Cl_2_N_2_O_2_Pt: H, 2.43%; N, 8.15%.

#### *cis*-Dichloro(diisopropylamine)-*trans*-dihydroxoplatinum(IV) (Iproplatin)

The synthesis was performed similarly to oxoplatin: *cis*-[PtCl_2_(ipa)_2_] (500 mg, 1.30 mmol) was suspended in 17 mL of H_2_O at 70 °C in the dark. Then, 22 mL of aqueous hydrogen peroxide (30% w/v) was added dropwise, and the mixture was stirred at the same temperature for 24 h. After that, the light-yellow solution was cooled at room temperature and exposed for several hours to light to eliminate the excess of hydrogen peroxide. Then, the volume was reduced *in vacuo* to induce crystallization of yellow crystals, which were solid filtrated and air-dried. Yield: 50%. ^1^H RMN (DMSO-*d*_6_, 400 MHz): δ 5.95 (s, 2H), 3.16 (sept, 1H, *J* = 6.3 Hz), 1.25 (d, 6H, *J* = 6.5 Hz). ^13^C RMN (DMSO-*d*_6_, 400 MHz): δ 46.5, 23.2. ^195^Pt RMN (DMSO-*d*_6_, 500 MHz): δ 954. IR (KBr, cm^–1^): 3500 ν(−OH); 3170, 3065 ν(−NH); 2980, 2870 ν(−CH/CH_3_); 1599 δ(−NH); 1120 δ(Pt–OH); 550 ν(Pt–N/Pt-O). Anal. Calcd for C_6_H_20_Cl_2_N_2_O_2_Pt: C, 17.23%; H, 4.82%; N, 6.70%. Found C_6_H_20_Cl_2_N_2_O_2_Pt: C, 17.46%; H, 4.58%; N,7.01%.

#### *cis*-Dichloro(diamine)-*trans*-[3-(triethoxysilyl)propylcarbamate]platinum(IV) (Pt(IV)-biSi-1)

The Pt(IV)-biSi-1 complex was synthesized as follows^30,31^: oxoplatin (300 mg, 0.90 mmol, 1 equiv) and 3-(triethoxysilyl)propyl isocyanate (900 μL, 3.6 mmol, 4 equiv) were added into 2.5 mL of anhydrous DMF under an inert atmosphere, and the mixture was stirred at room temperature overnight. The final suspension was filtrated, and the filtrate was completely removed under reduced pressure. The resulting yellow oil was washed with petroleum ether to eliminate the excess of isocyanate, stirring vigorously for 1 h. During the stirring, the oil became a pale-yellow solid that was filtrated and air-dried. Yield: 60%. ^1^H RMN (DMSO-*d*_6_, 400 MHz): δ 6.68 (s, 6H), 6.54 (s, 1H, major isomer, OCONH), 5.93 (s, 1H, minor isomer, OCONH), 3.72 (q, 12H, *J* = 7.5 Hz), 2.87 (m, 4H), 1.40 (m, 4H), 1.14 (t, 18H, *J* = 7.5 Hz), 0.49 (m, 4H). ^13^C RMN (DMSO-*d*_6_, 400 MHz): δ 164.2, 58.1, 44.4, 23.8, 18.7, 7.8. ^195^Pt RMN (DMSO-*d*_6_, 500 MHz): δ 1278 (major isomer) and 1264 (minor isomer). IR (KBr, cm^–1^): 3380, 3230 ν(−NH); 2975, 2928, 2887 ν(−CH/–CH2/CH_3_); 1636 ν(OCONH); 1078 ν(Si–OCH_2_CH_3_). MS [M + K^+^] = 867.2. Anal. Calcd for C_20_H_50_Cl_2_N_4_O_10_PtSi_2_: C, 28.98%; H, 6.08%; N, 6.76%. Found C_20_H_50_Cl_2_N_4_O_10_PtSi_2_: C, 28.55%; H, 6.26%; N, 6.89%. The purity of this compound (>95%) was confirmed by HPLC (Figure S8) and elemental analysis.

#### *cis*-Dichloro(diisopropylamine)-*trans*-[3-(triethoxysilyl)propylcarbamate]platinum(IV) (Pt(IV)-biSi-2)

The Pt(IV)-biSi-2 complex was synthesized similarly to Pt(IV)-biSi-1: iproplatin (300 mg, 0.72 mmol, 1 equiv) and 3-(triethoxysilyl)propyl isocyanate (718 μL, 2.88 mmol, 4 equiv) were added into 2.5 mL of anhydrous DMF under an inert atmosphere, and the mixture was stirred at room temperature overnight. The solvent was completely removed under reduced pressure, and the resulting yellow oil was washed with petroleum ether to eliminate the excess isocyanate, stirring vigorously for 1 h. During the stirring, the oil became a yellow crystalline solid that was finally air-dried. Yield: 68%. ^1^H RMN (DMSO-*d*_6_, 400 MHz): δ 7.80 (s, 4H), 6.84 (s, 1H, major isomer, OCONH), 6.52 (s, 1H, minor isomer, OCONH), 3.71 (q, 12H, *J* = 7.5 Hz), 3.28 (m, 1H), 2.89 (m, 4H), 1.39 (m, 4H), 1.20 (d, 12H, *J* = 7.5 Hz), 1.13 (t, 18H, *J* = 7.5 Hz), 0.49 (m, 4H). ^13^C RMN (DMSO-*d*_6_, 400 MHz): δ 165.5, 58.3, 47.5, 43.5, 23.0, 20.7, 18.7, 7.4. ^195^Pt RMN (DMSO-*d*_6_, 500 MHz): δ 1370 (major isomer) and 1355 (minor isomer). IR (KBr, cm^–1^): 3375 ν(−NH); 2973, 2933 ν(−CH/–CH2/CH_3_); 1640 ν(OCONH); 1080 ν(Si–OCH_2_CH_3_). MS [M^+^] = 913.5. Anal. Calcd for C_26_H_62_Cl_2_N_4_O_10_PtSi_2_: C, 34.21%; H, 6.85%; N, 6.14%. Found C_26_H_62_Cl_2_N_4_O_10_PtSi_2_: C, 34.50%; H, 6.68%; N, 6.15%. The purity of this compound (>95%) was confirmed by HPLC (Figure S11) and elemental analysis.

### ^195^Pt NMR Kinetic Studies Using Reducing Agents

Ten milligrams of each complex (Pt(IV)-biSi-1 and Pt(IV)-biSi-2) was first dissolved in 300 μL of DMSO-*d*_6_, stirring the mixture vigorously at 37 °C. Each solution was then slowly added to 100 μL of a solution of the corresponding reducing agent (AsA or GSH in an excess of 4:1 against each complex) in D_2_O at 37 °C. After that, 100 μL of DMSO-*d*_6_ was added carefully to each mixture, reaching a final concentration of 4:1 (DMSO-*d*_6_/D_2_O). All of these mixtures were performed using a thermoshaker. Each sample was monitored by ^195^Pt NMR at 2, 6, and 24 h.

### Determination of the Purity of the Pt(IV)-biSi-1 and -2 Complexes by HPLC

Samples of both Pt(IV) bis-organosilane complexes were prepared from a 1 mM stock solution in DMSO to reach a final concentration of 10^–4^ mM by diluting with Milli-Q water, achieving a final volume of 2 mL with 2% of DMSO. Each solution was analyzed by UHPLC-HESI-MS/MS on an Agilent 1200 system using an Intensity Solo 2 C18 column (100 × 2.1 mm): flow rate, 0.4 mL min^–1^; gradient solvent system A/B (water/methanol), initial 80% A + 20% B; 21.8 min linear gradient to 80% A + 20% B; 9.2 min linear gradient to 100% B.

### Cell Culture

HIEC6 human healthy intestinal cells and HCT 116 and HT-29 human colon cancer cells were supplied by ATCC. Colon cancer cells were cultured in Dulbecco’s modified Eagle’s medium (DMEM) for HCT 116 and HT-29 supplemented with fetal bovine serum (10%). HIEC6 cells were cultured in Opti-MEM supplemented with fetal bovine serum (4%), HEPES (20 mM), glutamine (10 mM), and epidermal growth factor (10 ng/mL). Cells were maintained at 37 °C in a humidified atmosphere containing 5% CO_2_. Cells were seeded at 30% confluence in DMEM for HCT 116 and in Opti-MEM for HIEC6; after cell adhesion, HIEC6 cells were cultured in DMEM for 24h before being treated. All cells were treated with the indicated components at concentrations of 0.25–50 μM and incubated for 1–5 days as specified. The complexes were freshly prepared in DMSO (except cisplatin, which was directly diluted in Milli-Q H_2_O) and diluted with DMEM to the desired concentration.

### Cell Viability by MTT (3-[4,5-Dimethylthiazole-2-yl]-2,5-diphenyltetrazolium bromide) Assay

The MTT assay relies on a colorimetric reaction based on the capacity of living cells to reduce MTT to formazan and change the color to purple, which is easily measured by colorimetry. This assay estimates the cellular viability after the indicated treatments. Cells were treated with MTT (1:10 in culture medium) and incubated at 37 °C for 3 h for HCT 116 and HT-29 or 6 h for HIEC6. After removal of the medium, the formazan was resuspended in DMSO, transferred to p96 plates, and analyzed by a SpectraFLUOR (Tecan) at 542 nm. In all cases, viability was measured in duplicates of each point in three independent experiments.

### Trypan Blue Exclusion Test

The trypan blue exclusion test estimates cytotoxicity (or viability) because intact cell membranes exclude trypan blue, whereas dead cells do not. HCT 116 cells were harvested by trypsinization followed by centrifugation at 500*g* for 5 min. The cell pellet was resuspended in 1 mL of PBS. Equal volumes of trypan blue (Bio-Rad cat. no. 1450021) 0.4% and cell suspension were mixed and incubated for 1–3 min at room temperature, and cell counting was carried out using a TC20 Automated Cell Counter (Bio-Rad cat. no. 1450102). The percentage of viable cells was calculated by dividing the number of viable cells per milliliter by the number total of cells per milliliter) × 100. Representative images were acquired using a Zeis light microscope with a 20× objective.

### Cell Viability by Flow Cytometry

Cell were trypsinized, centrifuged at 500*g* for 5 min, resuspended in PBS, and stained with 7-aminoactinomycin D (7AAD, Santa Cruz Biotechnology), a DNA intercalating dye excluded from viable cells due to membrane impermeability. The 7AAD was excited at 546 nm and analyzed at 656 nm. Only apoptotic and necrotic cells are stained by these dyes. After incubation for 50 min in the dark at 37 °C, the cells were analyzed by flow cytometry (CytoFlex, Beckman Coulter). The percentages of cells stained with or without dyes were analyzed using the CytExpert Software (Beckman Coulter).

### Stability of the Pt(IV) Final Complexes by ^195^Pt NMR

Eight milligrams of each complex (Pt(IV)-biSi-1 and Pt(IV)-biSi-2) was first dissolved in a mixture of 100 μL of DMSO-*d*_6_ and 100 μL of DMEM, stirring vigorously at 37 °C. To each solution was then slowly and carefully added 300 μL of DMEM to avoid the precipitation of the complex, reaching a final concentration of 1:4 (DMSO-*d*_6_/DMEM). All these mixtures were performed using a thermoshaker. Each sample was monitored by ^195^Pt NMR at 0, 24, 48, and 120 h.

### Platinum Internalization in HCT 116 Cells

The internalization of platinum in HCT 116 cells was determined by the inductively coupled plasma optical emission spectroscopy (ICP-OES) method. First, HCT 116 cells were cultivated in Dulbecco’s modified Eagle’s medium (DMEM) in a similar way to the cell culture experiments. Cells were maintained at 37 °C in a humidified atmosphere containing 5% CO_2_. Cells were seeded at 30% confluence in DMEM in a p100 plate. All cells were treated with the complexes Pt(IV)-biSi-1 and Pt(IV)-biSi-2 and cisplatin as reference at concentrations of 5 and 25 μM (separately) and incubated for 0, 12, 24, and 48 h. The complexes were freshly prepared in DMSO (except cisplatin, which was directly diluted in Milli-Q H_2_O) and diluted with DMEM to the desired concentration. After each time, cells were extracted with trypsin and subsequently centrifuged to separate the pellet and the supernatant. For the ICP measurements, the preparation of samples for the platinum determination was as follows^72^: 1 mL of Milli-Q H_2_O was added to each pellet of cells, and they were sonicated for 5 min at rt. Then, 1.5 mL of concentrated HNO_3_ was added to each suspension, and it was heated for 3 h at 70 °C. After the acidic digestion, 7.5 mL of Milli-Q H_2_O was added to each suspension to reach a final volume of 10 mL. At the same time, a pattern curve was prepared using the K_2_PtCl_4_ salt. Finally, the amount of platinum in the digested samples, together with the platinum diluted in the supernatant, was determined by ICP-OES.

### Western Blot

Cells were washed with iced PBS before extract preparation and scraped in whole-cell protein lysis buffer (62.5 mM Tris-HCl pH 6.8, 2% sodium dodecyl sulfate (SDS), 0.01% bromophenol blue sodium salt, and 10% glycerol). Whole-cell protein lysates were incubated 15 min at 95 °C and directly used as whole cell extract or frozen at −80 °C. Proteins from lysed cells or immunoprecipitates were denatured and loaded on sodium dodecyl sulfate polyacrylamide gels and then transferred to polyvinylidene difluoride membranes (Bio-Rad). After blocking with 5% (w/v) milk, the membrane was incubated with the corresponding primary antibodies, anti-p53 sc-126 (Santa Cruz Biotechnology) or anti-tubulin (Sigma-Aldrich), and secondary antibodies (Bio-Rad). The specific bands were analyzed using the ChemiDoc Imaging Systems (Bio-Rad).

### Immunofluorescence

Cells in coverslips were washed three times and fixed with 4% paraformaldehyde in PBS (pH 7.4) for 10 min, washed again, permeabilized (PBS [pH7.4], 0.5% Triton X-100, 0.2% BSA) for 5 min, blocked (PBS [pH7,4], 0,05% Triton X-100, 5% BSA) for 1 h at room temperature, and incubated with primary antibody overnight at 4 °C. After three washes (5 min), cells were incubated with secondary antibody for 1 h at room temperature, and the nuclei were stained with 4,6-diamidino-2-phenylindole (DAPI, 1:10,000, Invitrogen) for 10 min. Slides were mounted, and images were acquired using an FV3000 confocal microscope (Olympus) with a 63× objective. The antibodies rabbit monoclonal anti-Histone H2A.X (D17A3) (dilution 1:400) and mouse monoclonal anti-Lamin A/C (4C11) (dilution 1:400) were purchased from Cell Signaling (Danvers, Massachusetts, USA). Donkey Anti-Rabbit-Alexa Fluor 488 (dilution 1:500) and Donkey Anti-Mouse-Alexa Fluor 594 (dilution 1:500) were purchased from Invitrogen (Barcelona, Spain).

### *InVivo* Experiments

Animal experiments were reviewed and approved by the Research Ethics and Animal Welfare Committee of Instituto de Salud Carlos III, Madrid (PROEX 198-18) and performed according to the Spanish Policy for Animal Protection RD53/2013 and the European Union Directive 2010/63 regarding the protection of animals destined for experimental and other scientific purposes.

To establish the subcutaneous tumor, 2 × 10^6^ HCT 116 colon carcinoma cells were resuspended 1:1 in culture media and Matrigel (BD) and injected subcutaneously. Six to eight week old female athymic nude Foxn1nu mice (Harlan Iberica) were used for each point. When tumors were visible (average volume of 30 mm^3^), animals started receiving either Pt(IV)-biSi-2 or vehicle (PBS) through intraperitoneal (i.p.) injection (total volume 200 μL). Animals receiving PBS and cisplatin (a clinically approved drug) were used as untreated and positive controls, respectively, to compare the effectiveness of Pt(IV)-biSi-2 based therapy. Two therapeutic approaches were compared: acute and chronic (see diagram in Figure 5). Acute treatment was performed (three injections of 10 mg/kg); chronic treatment relied on injections of 1 mg/kg every 3–4 days. The antitumor potential of Pt(IV)-biSi-2 was evaluated by measuring tumor growth in each group with a caliper every 2 to 3 days for 4 weeks. Tumor growth was monitored by personnel unaware of the group distribution. Experiments were finalized when control animals achieved the maximum tumor size approved by the Ethical Committee. Animals were sacrificed by euthanasia by CO_2_ inhalation. Selected organs were collected from each animal for histopathological analysis. Samples were fixed in 4% paraformaldehyde for 2 h followed by cold 70% ethanol. Plasma serum was collected to determine hepatic and renal toxicity of treatments using a SPIN200E automatic bioanalyzer (Spinreact).

### Statistical Analysis

The results are presented as fold induction. Values are mean ± SEM with reference to untreated cells from three independent biological replicas. Multiple comparisons were performed using ANOVA with Bonferroni’s *post hoc* test. Differences were considered statistically significant if *p* ≤ 0.05.

## Supporting Information Available

Molecular strings and *in vitro* data (CSV)
Details about complete experimental procedures and supporting figures (PDF)

## ABBREVIATIONS

Pt: platinum (Pt)
ipa: (isopropylamine)
NMR: nuclear magnetic resonance
FTIR: Fourier transform infrared spectroscopy
ESI-MS: electrospray ionization mass spectrometry
HPLC: high-performance liquid chromatography
STEM: scanning transmission electron microscopy
EDX: energy dispersive X-ray spectroscopy
AsA: ascorbic acid
GSH: glutathione
HCT 116: human colon cancer 116 cell line
HT-29: human colon adenocarcinoma cell line
HIEC6: nontumorigenic intestinal cell line
MTT: 3-[4,5-dimethylthiazole-2-yl]-2,5-diphenyltetrazolium bromide
IPC-OES: inductively coupled plasma optical emission spectroscopy
DAPI: 4′,6-diamidino-2-phenylindole
H2AX: histone H2A variant
