# Supplementary material for: Promising Anticancer Prodrugs Based on Pt(IV) Complexes with Bis-organosilane Ligands in Axial Positions
Source: J Med Chem. 2024 Apr 9;67(8):6410–24. doi: 10.1021/acs.jmedchem.3c02393 (PMC11056991; doi:10.1021/acs.jmedchem.3c02393)
Supplement: Supplementary file 2 — jm3c02393_si_002.pdf [file jm3c02393_si_002.pdf]

SUPPORTING INFORMATION

Promising *anticancer prodrugs based on Pt(IV) complexes with bis-organosilane ligands in axial positions*

Francisco Navas[a], Ana Chocarro-Calvo[b], Patricia Iglesias-Hernández[c], Paloma Fernández-García[a], Victoria Morales[a], José Manuel García-Martínez[b], Raúl Sanz[a], Antonio De la Vieja[c]*, Custodia García-Jiménez[b]*, Rafael A. García-Muñoz[a]*.

[a] Group of Chemical and Environmental Engineering, Rey Juan Carlos University. C/Tulipán s/n, 28933 Móstoles, Madrid, Spain. E-mail: rafael.garcia@urjc.es

[b] Department of Basic Health Sciences. Rey Juan Carlos University. Avda. Atenas s/n, 28922 Alcorcón, Madrid, Spain. E-mail: custodia.garcia@urjc.es

[c] Endocrine Tumor Unit Chronic Disease Program (UFIEC). Carlos III Health Institute. Ctra. Majadahonda a Pozuelo km 2,2. 28220 Majadahonda, Madrid, Spain. E-mail: adelavieja@isciii.es

Table of Contents

Figures S1-S13 S2-S16

Scheme S1 S17

Table 1 S17

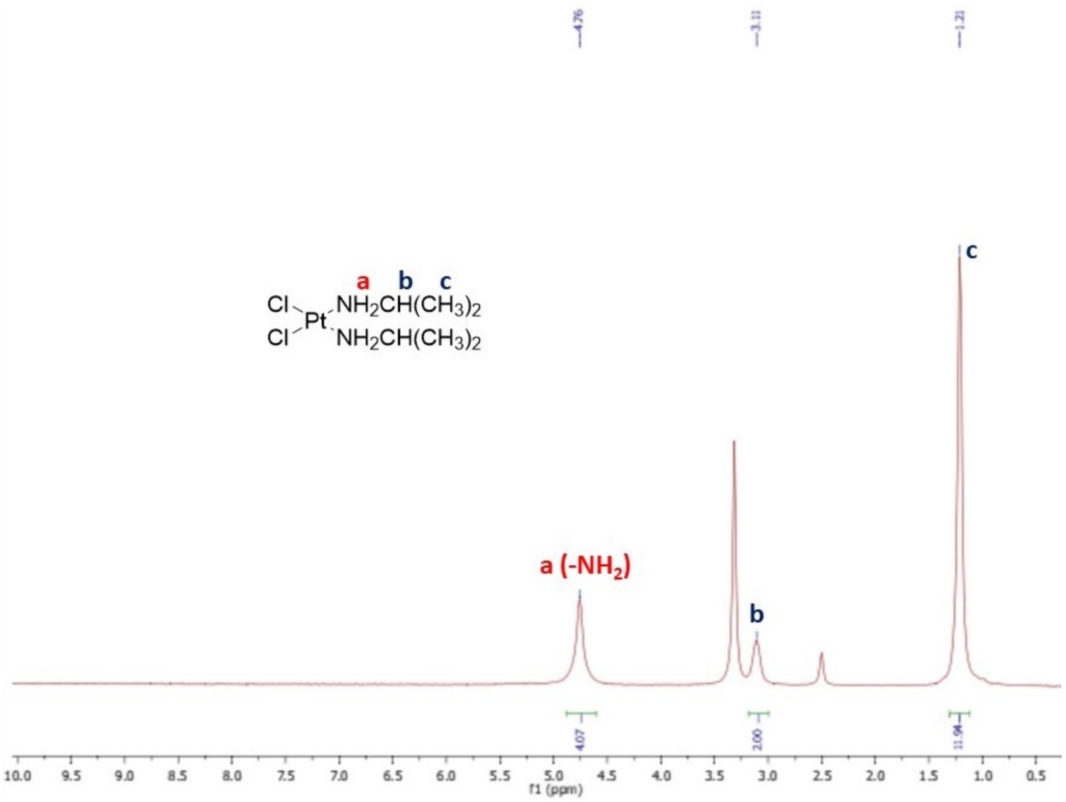
4.76
3.11
1.21
a b c
Cl Pt NH2CH(CH3)2
Cl NH2CH(CH3)2
c
a (-NH2)
b
4.07
2.00
11.94
10.0 9.5 9.0 8.5 8.0 7.5 7.0 6.5 6.0 5.5 5.0 4.5 4.0 3.5 3.0 2.5 2.0 1.5 1.0 0.5
f1 (ppm)

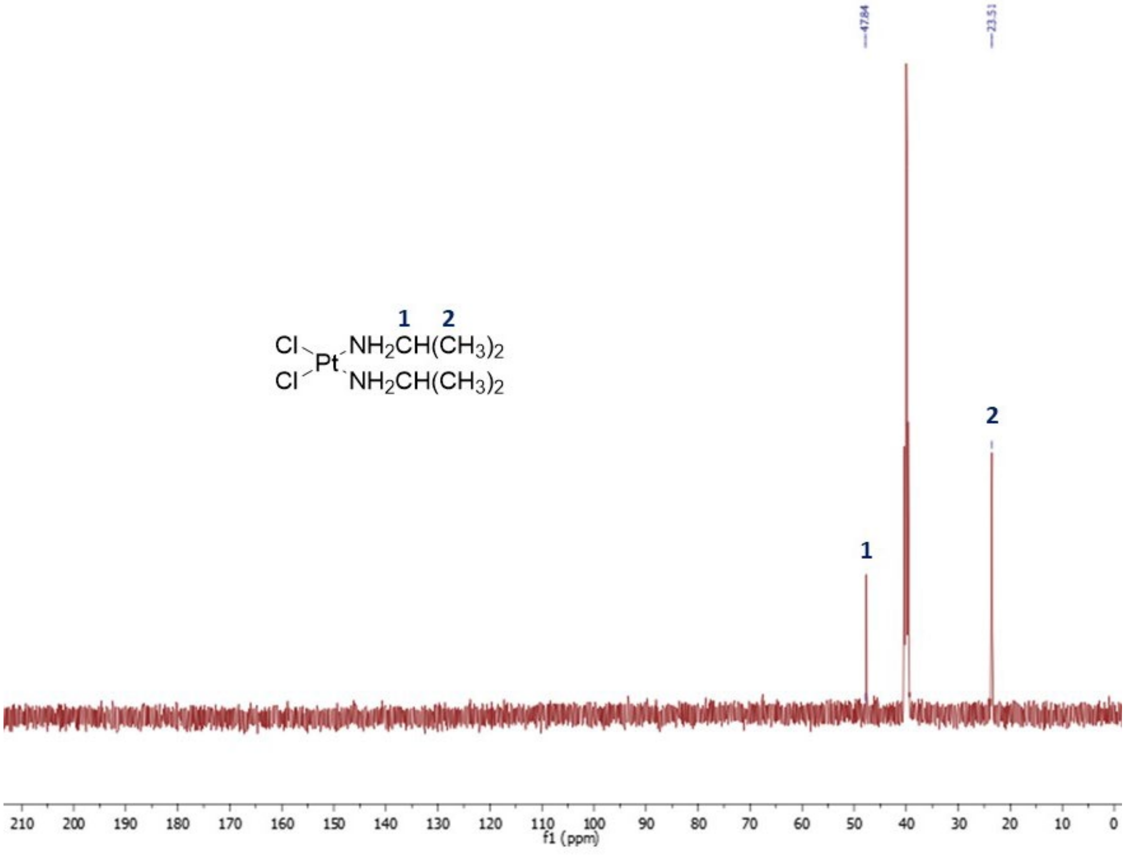
47.84
23.51
1 2
Cl Pt NH2CH(CH3)2
Cl NH2CH(CH3)2
2
1
210 200 190 180 170 160 150 140 130 120 110 100 90 80 70 60 50 40 30 20 10 0
f1 (ppm)

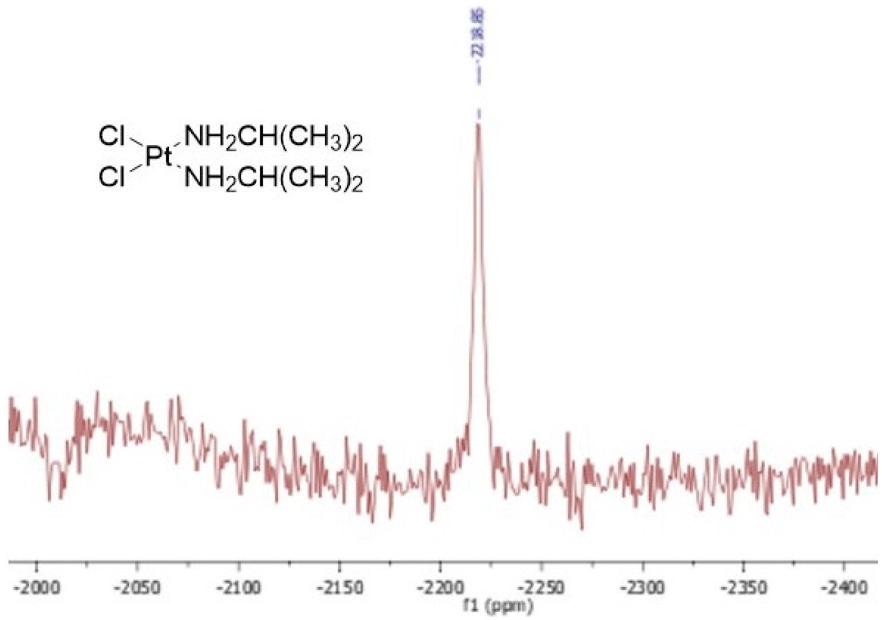

Figure S1. ^{1}H NMR, ^{13}C NMR and ^{195}Pt NMR spectra of *cis*-[Pt(ipa)$_2$Cl$_2$] in DMSO-d_6.

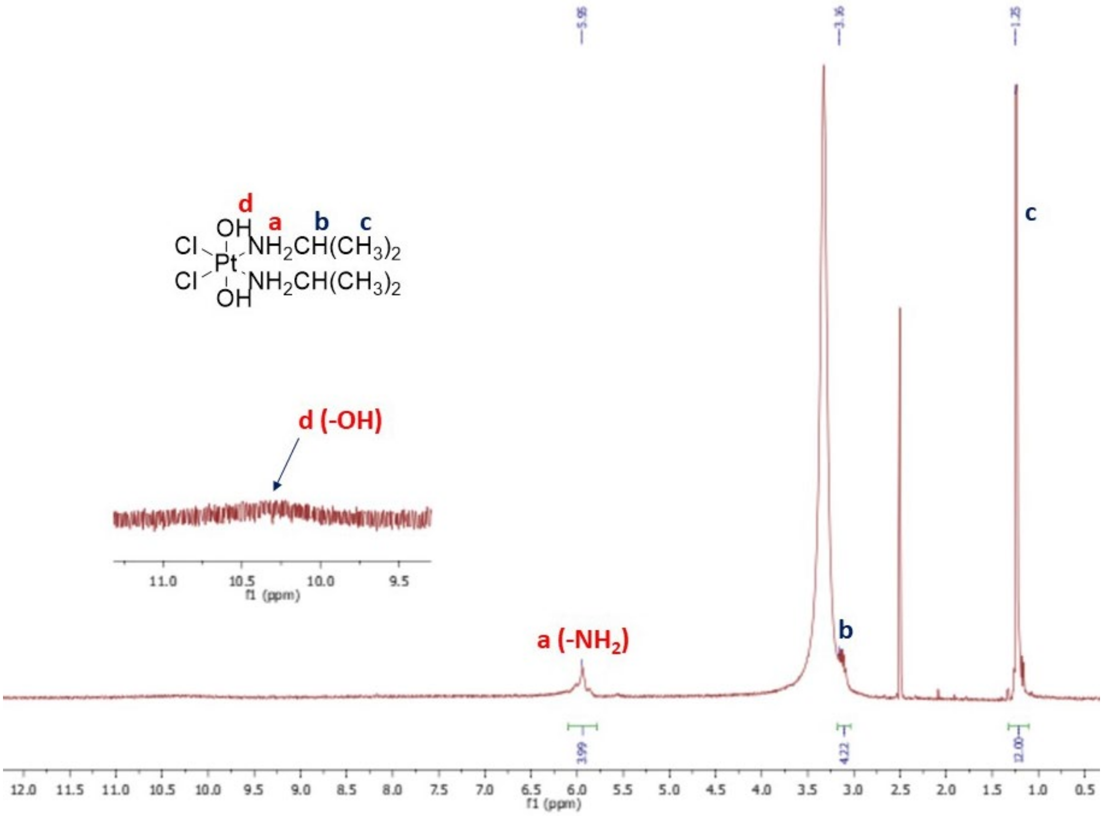

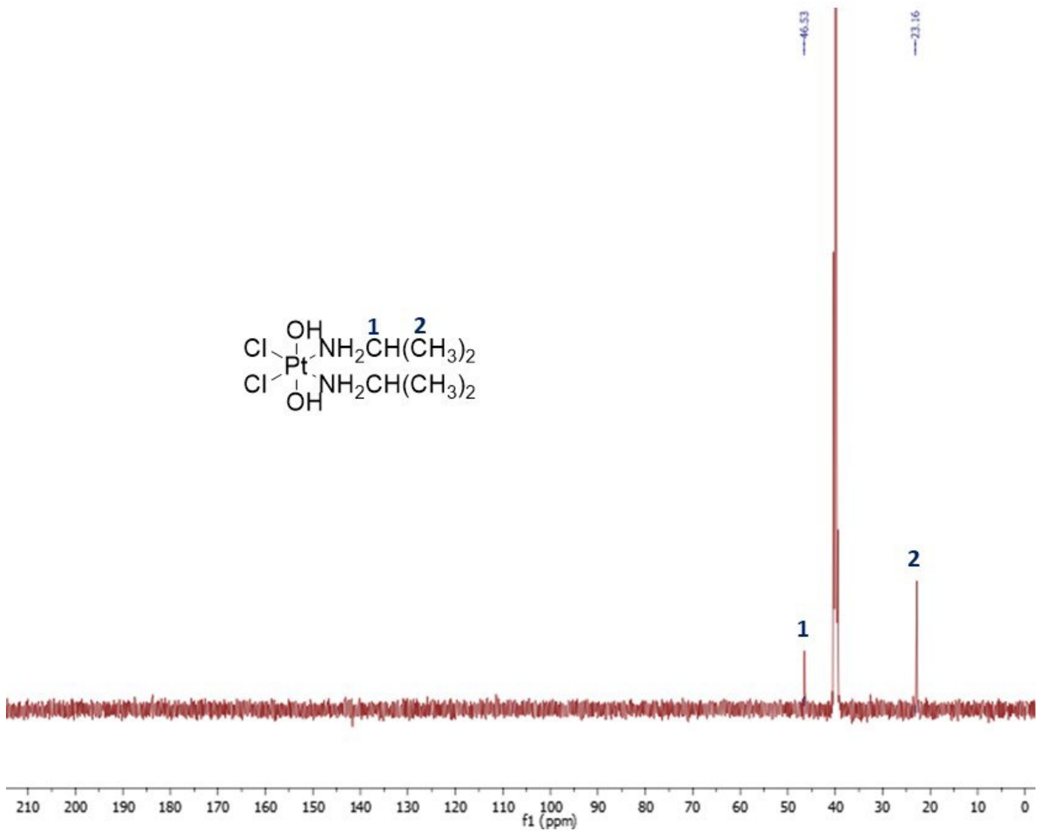

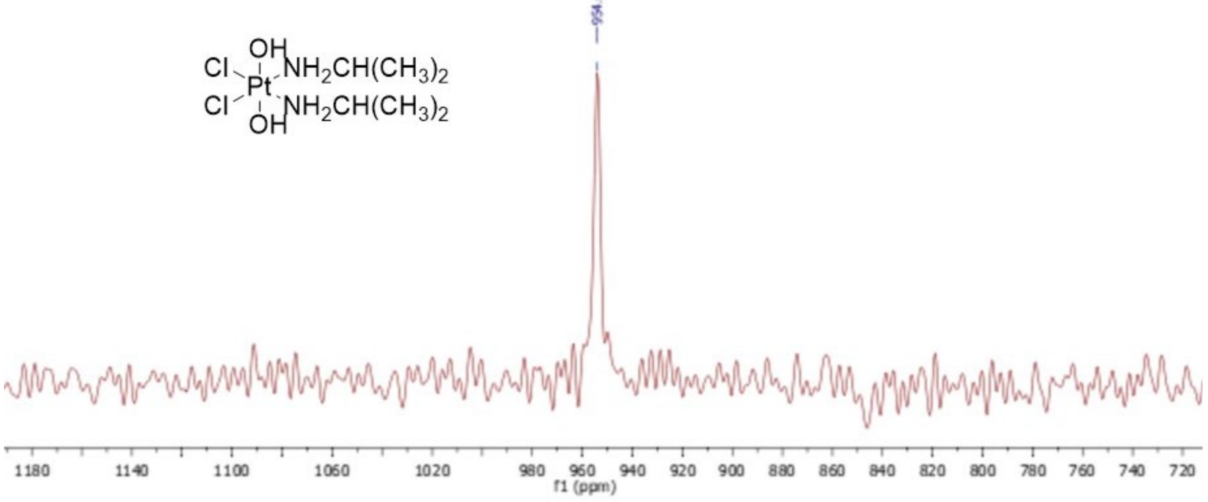

Figure S2. ^{1}H NMR, ^{13}C NMR, ^{195}Pt NMR spectra of iproplatin in DMSO-d_6.

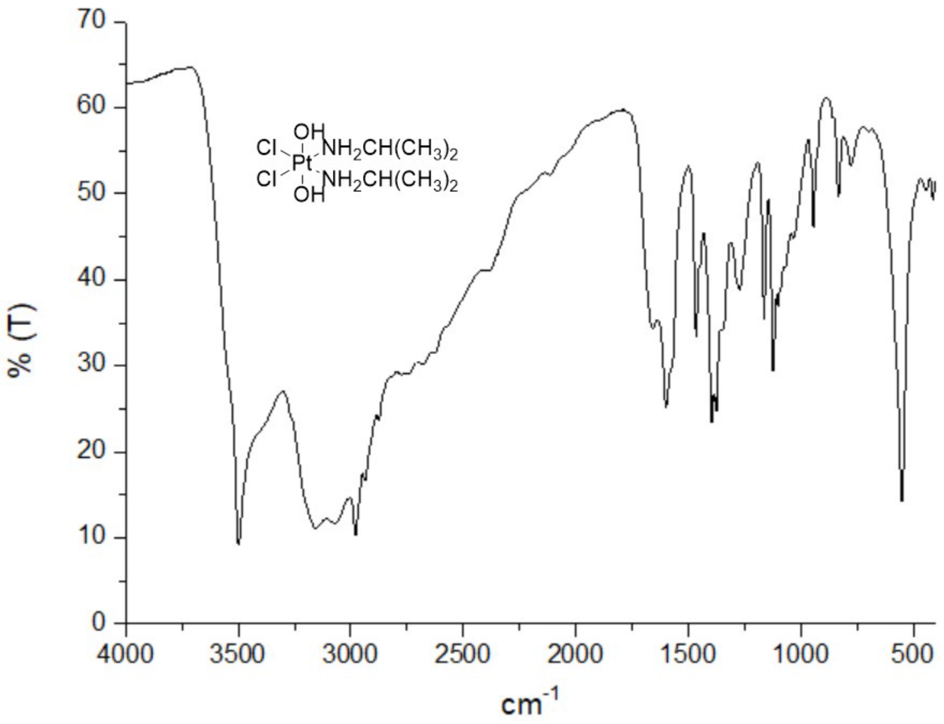

Figure S3. FTIR spectrum of iproplatin.

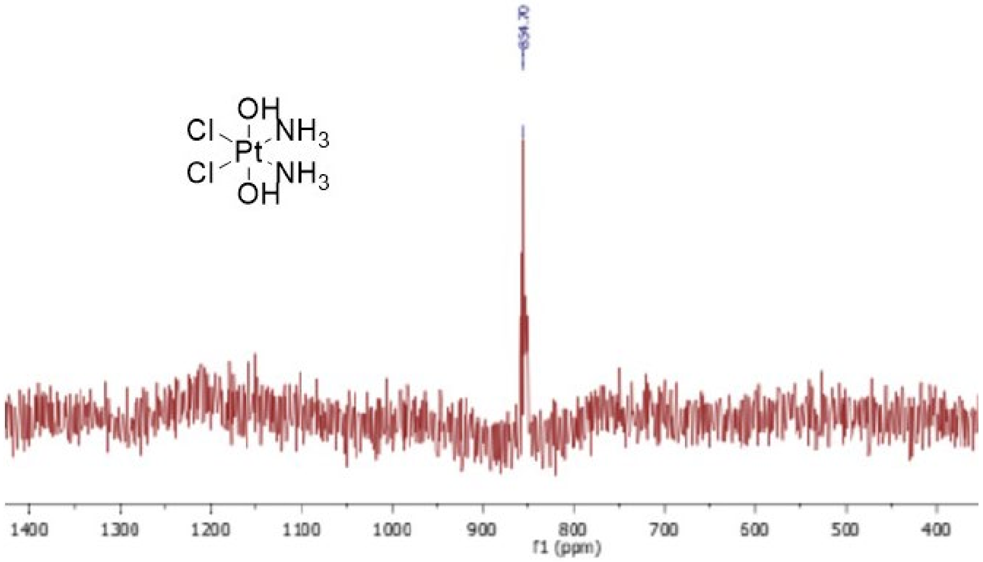

Figure S4. ^{195}Pt NMR spectrum of oxoplatin in D_2O.

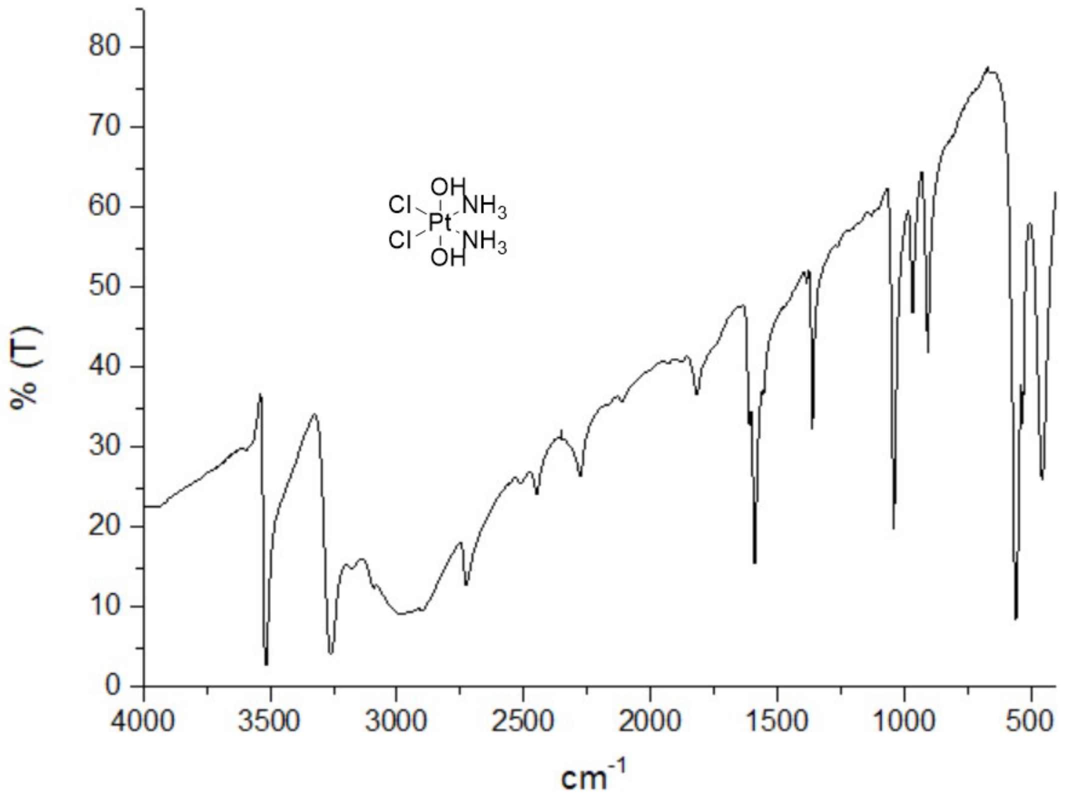

Figure S5. FTIR spectrum of oxoplatin.

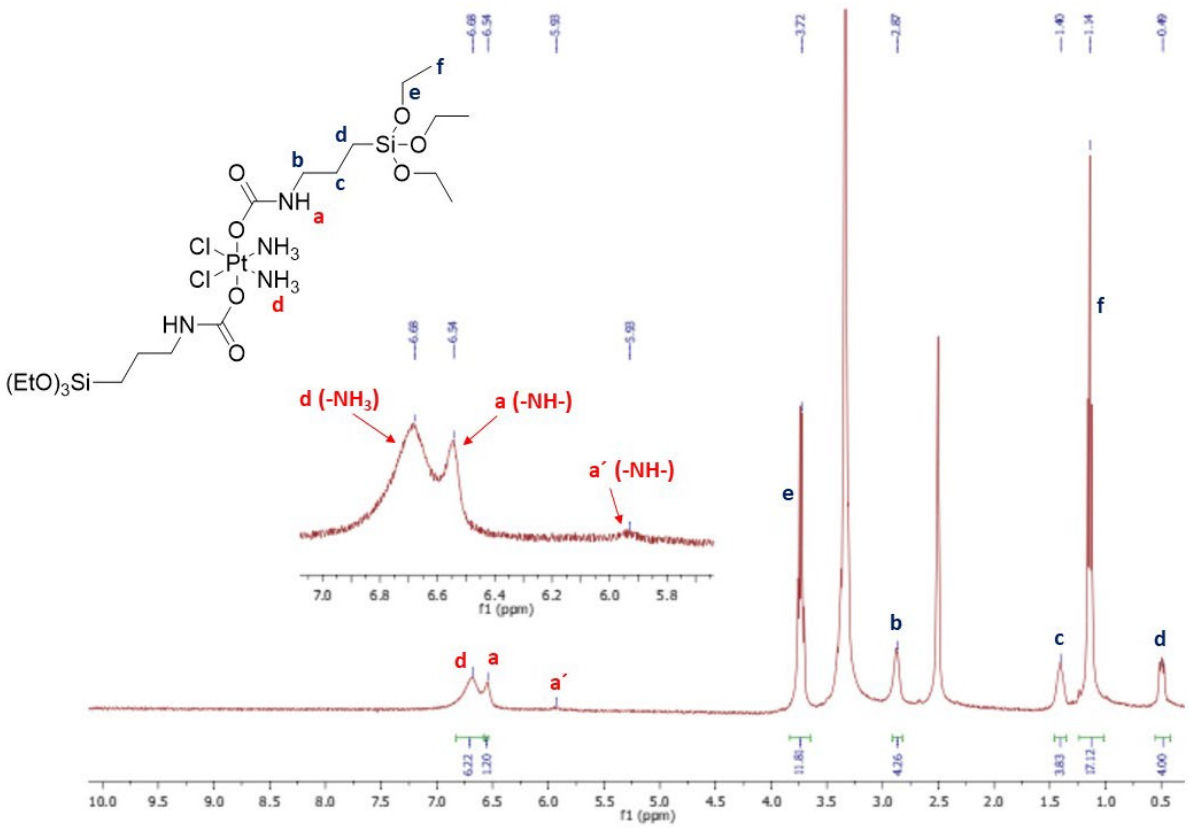
d (-NH3)
a (-NH-)
a´ (-NH-)
a
b
c
d
e
f
f1 (ppm)

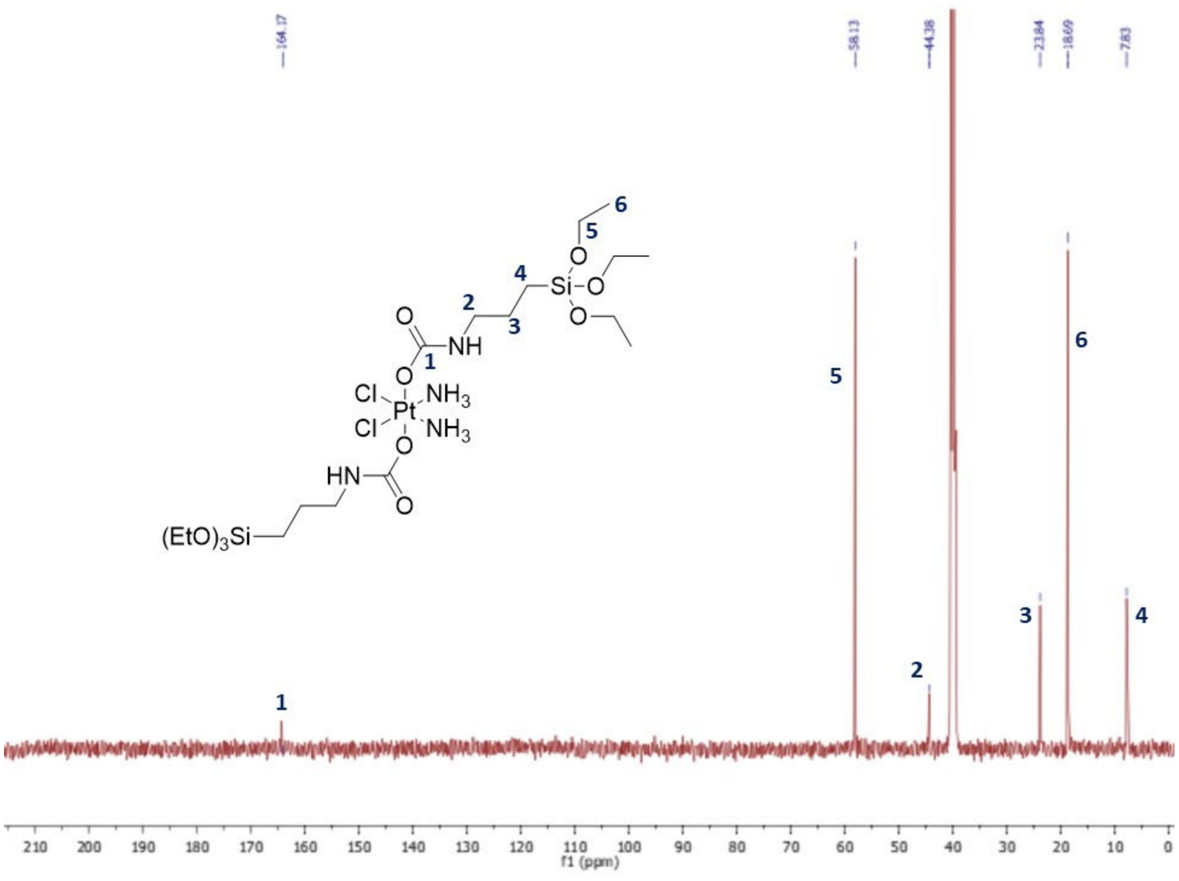
1
2
3
4
5
6
f1 (ppm)

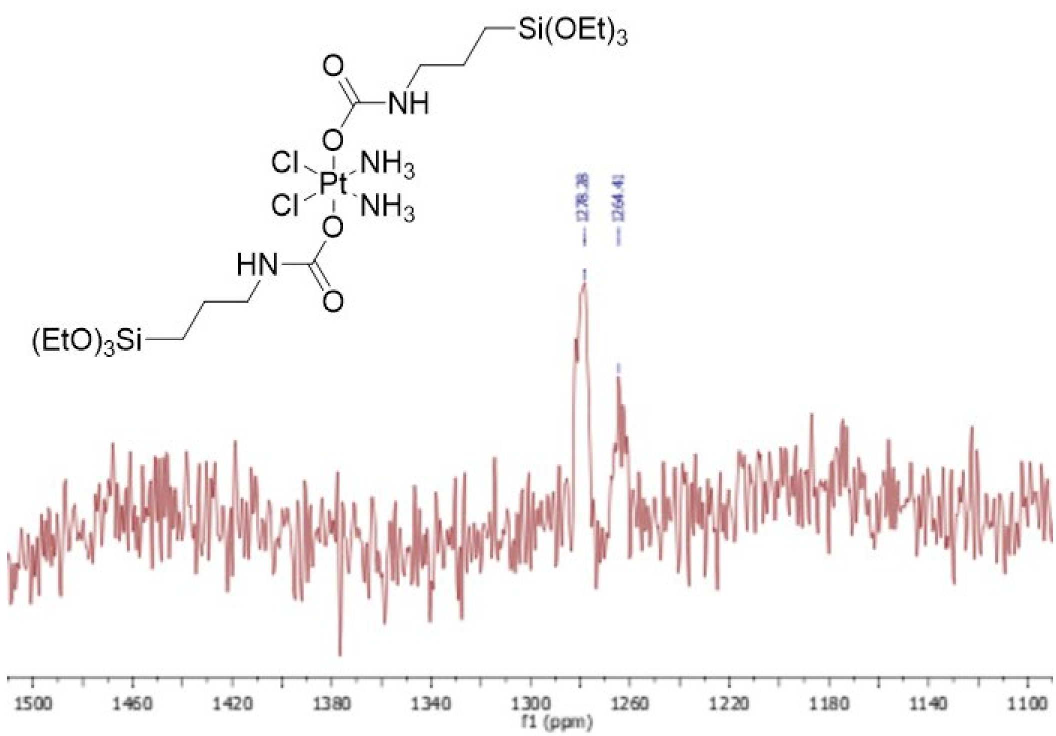

Figure S6. ^{1}H NMR, ^{13}C NMR and ^{195}Pt NMR spectra of Pt(IV)-biSi-1 in DMSO-d_6.

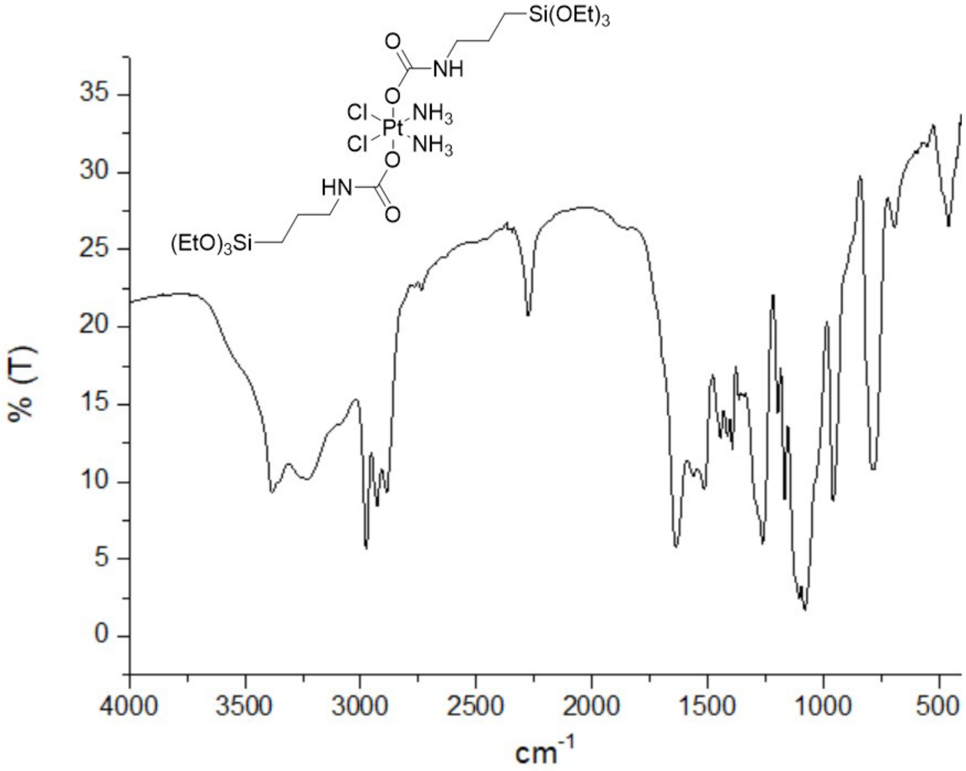

Figure S7. FTIR spectrum of Pt(IV)-biSi-1.

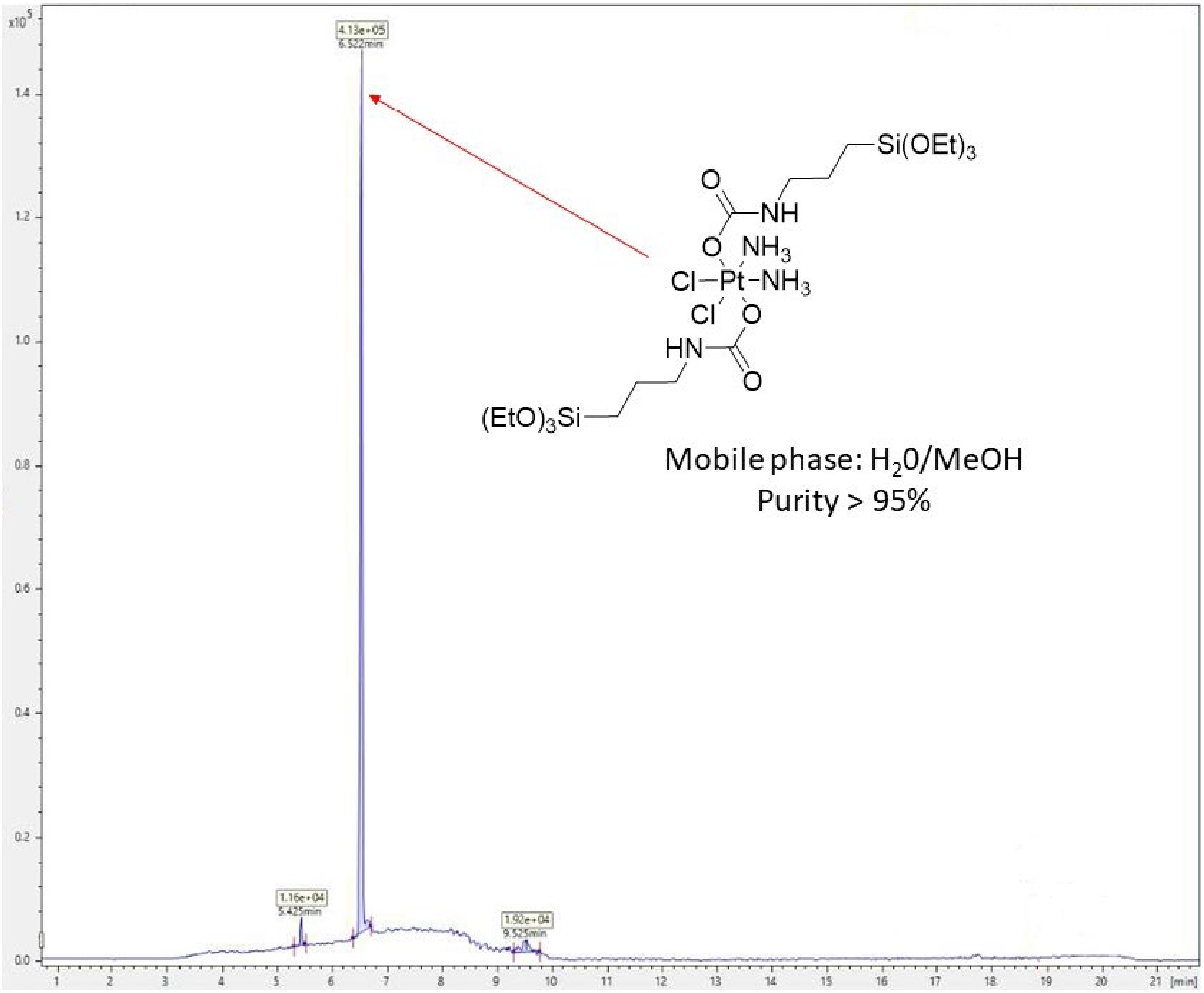

Figure S8. HPLC chromatogram of Pt(IV)-biSi-1.

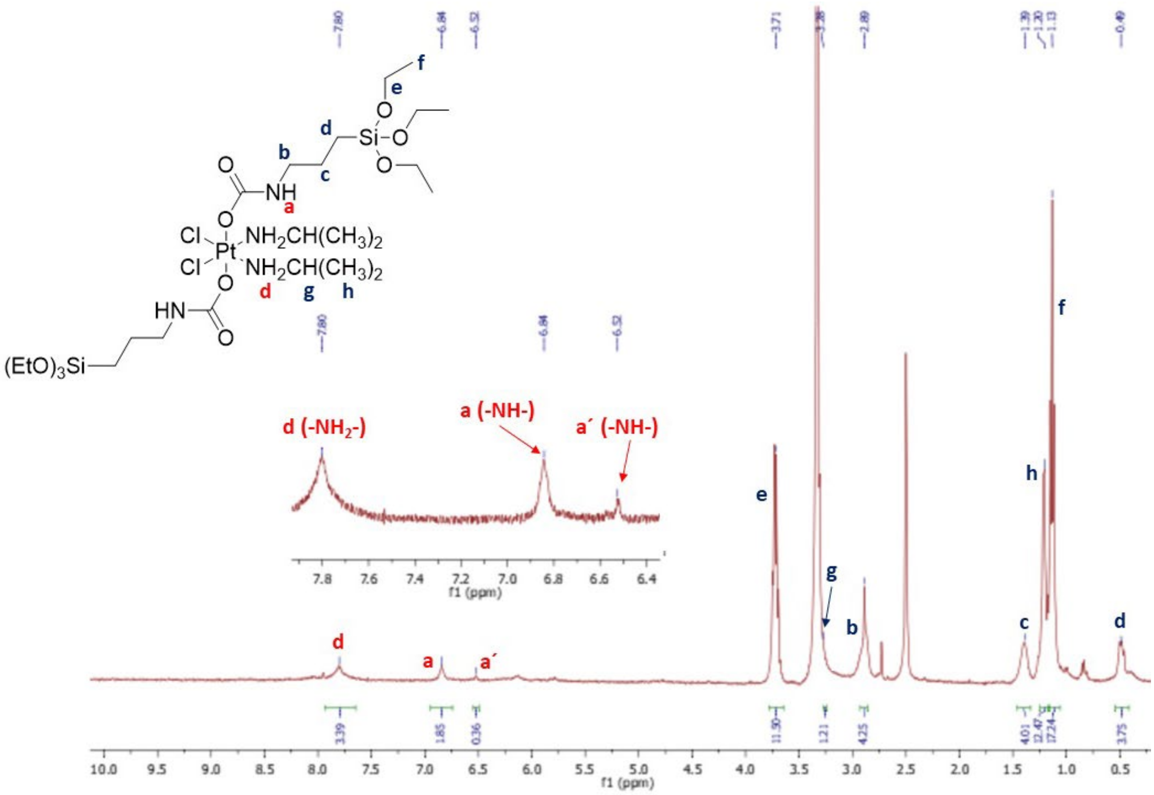
d (-NH$_2$-)
a (-NH-)
a´ (-NH-)
NH$_2$CH(CH$_3$)$_2$
NH$_2$CH(CH$_3$)$_2$
(EtO)$_3$Si
7.80
6.84
6.52
3.71
3.28
2.89
1.39
1.20
1.13
0.49
3.39
1.85
0.36
11.90
1.21
4.25
4.01
12.47
17.24
3.75
f1 (ppm)

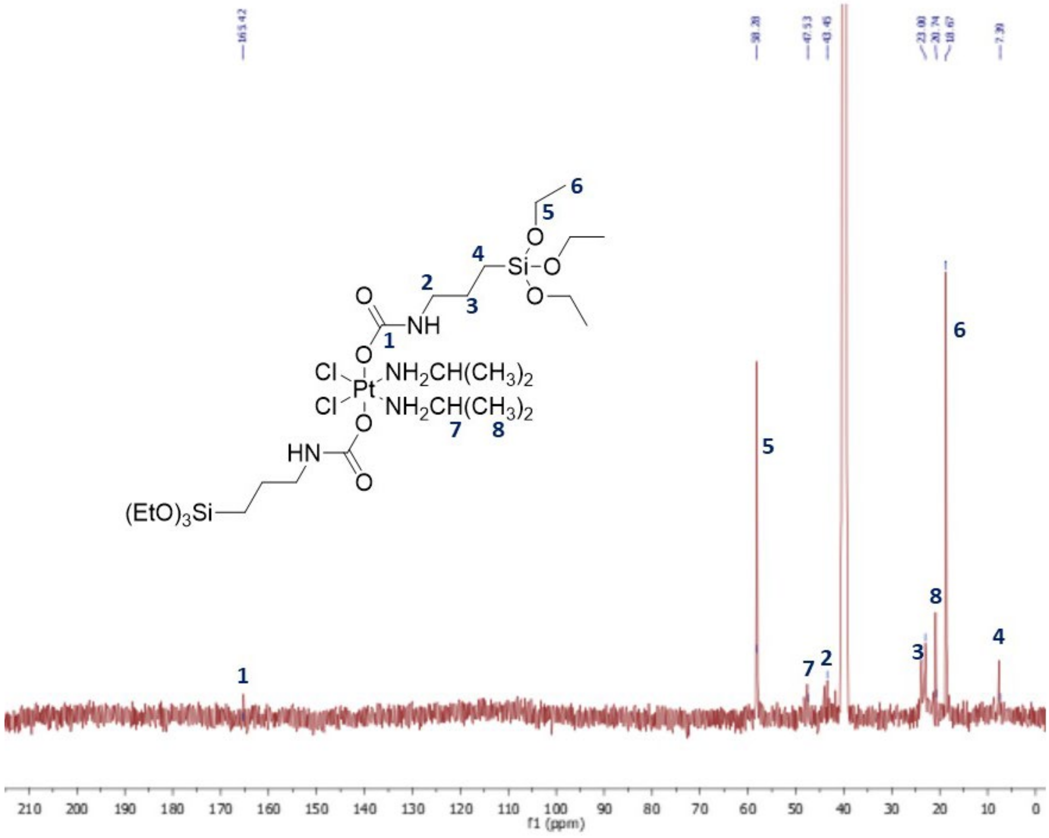
NH$_2$CH(CH$_3$)$_2$
NH$_2$CH(CH$_3$)$_2$
(EtO)$_3$Si
165.42
58.28
47.53
43.45
23.00
20.74
18.67
7.39
f1 (ppm)

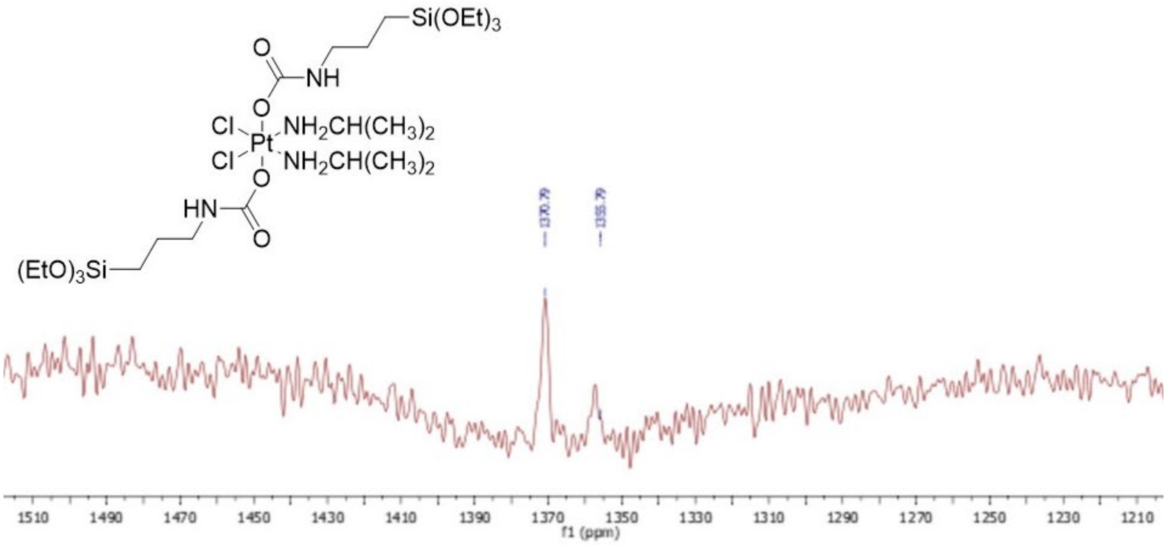

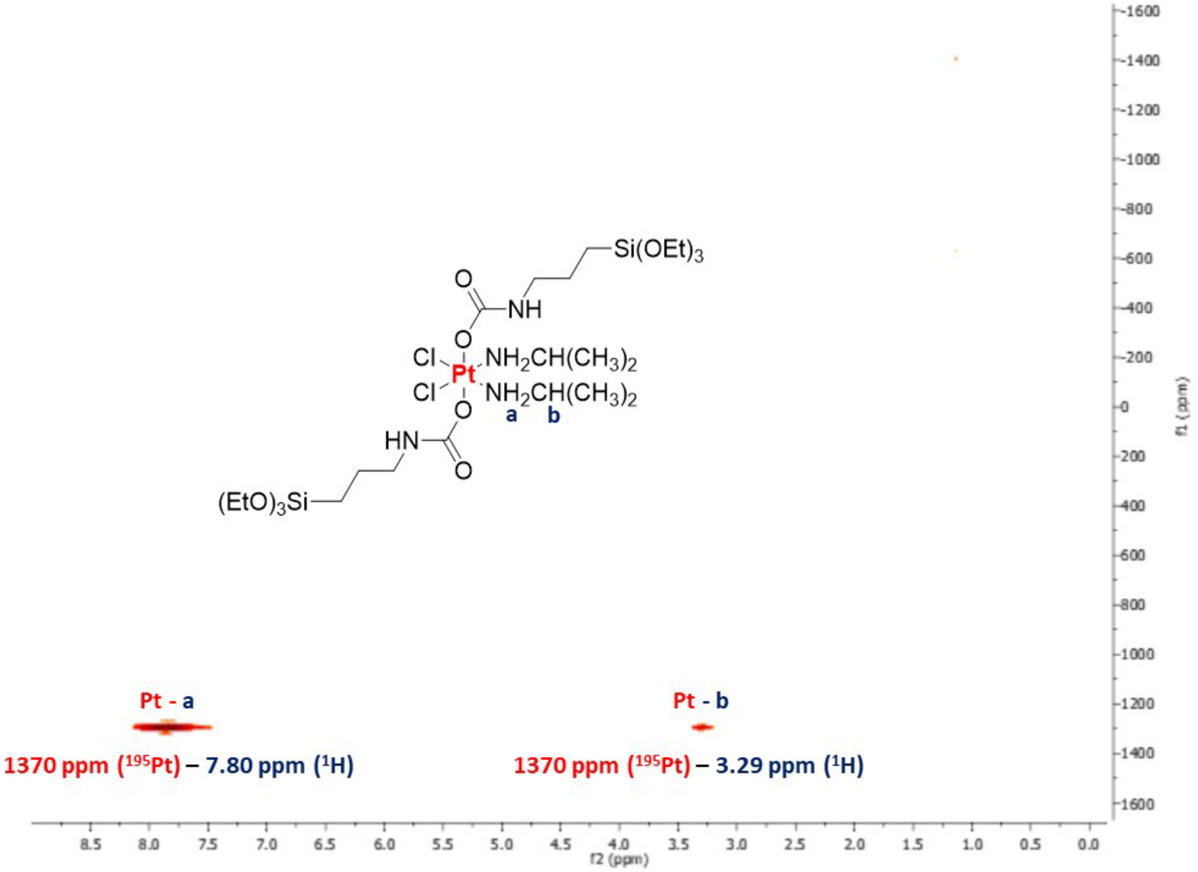

Figure S9. ^{1}H NMR, ^{13}C NMR, ^{195}Pt NMR and ^{1}H-^{195}Pt NMR spectra of Pt(IV)-biSi-2 in DMSO-d_6.

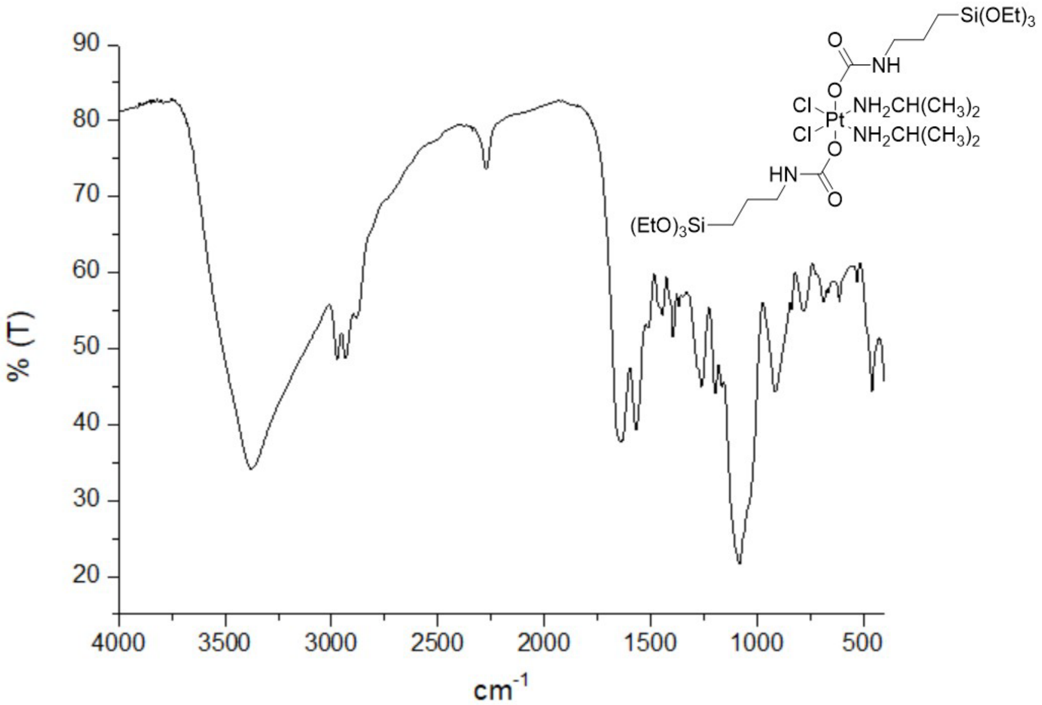

Figure S10. FTIR spectra of Pt(IV)-biSi-2.

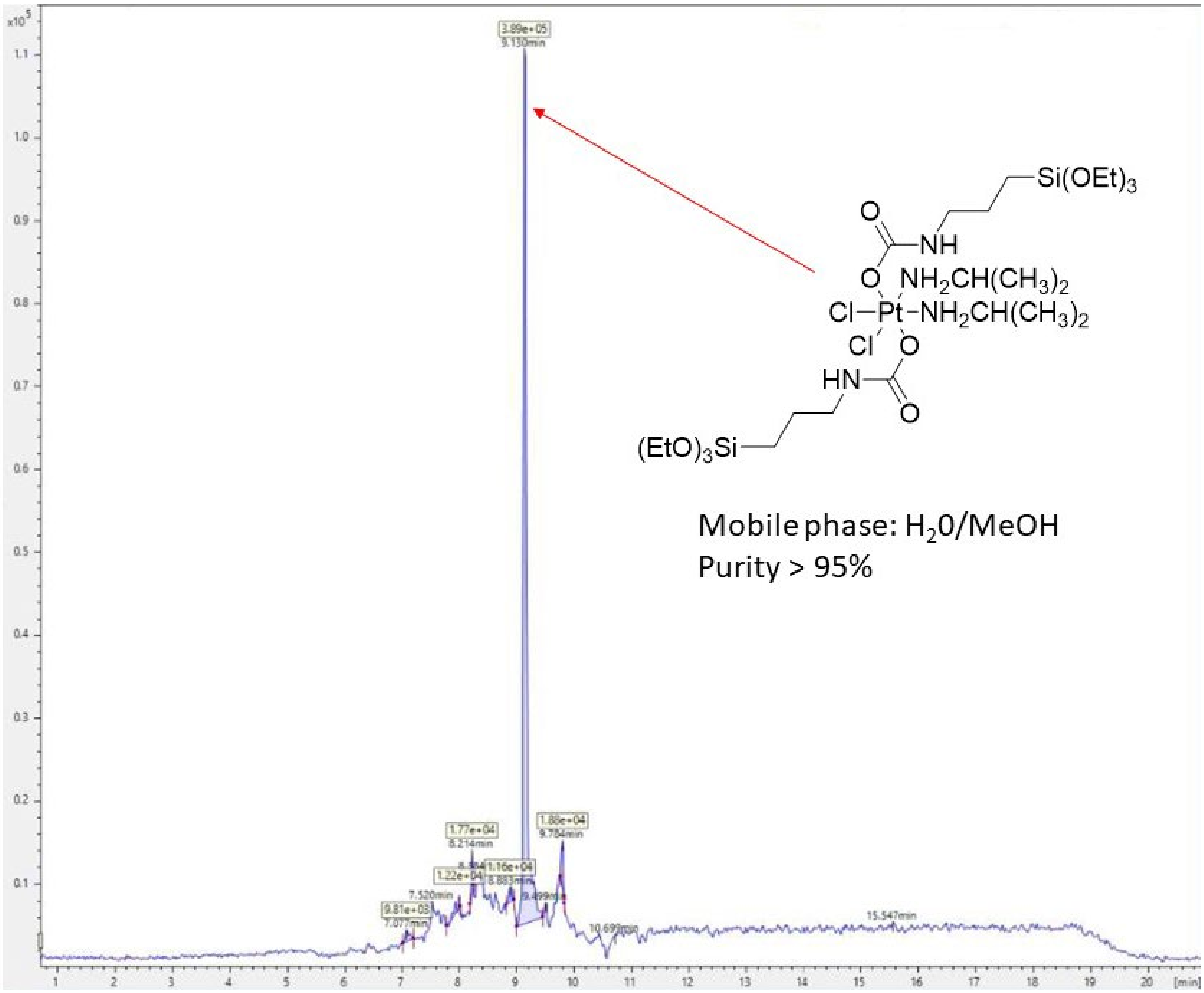

Figure S11. HPLC chromatogram of Pt(IV)-biSi-2.

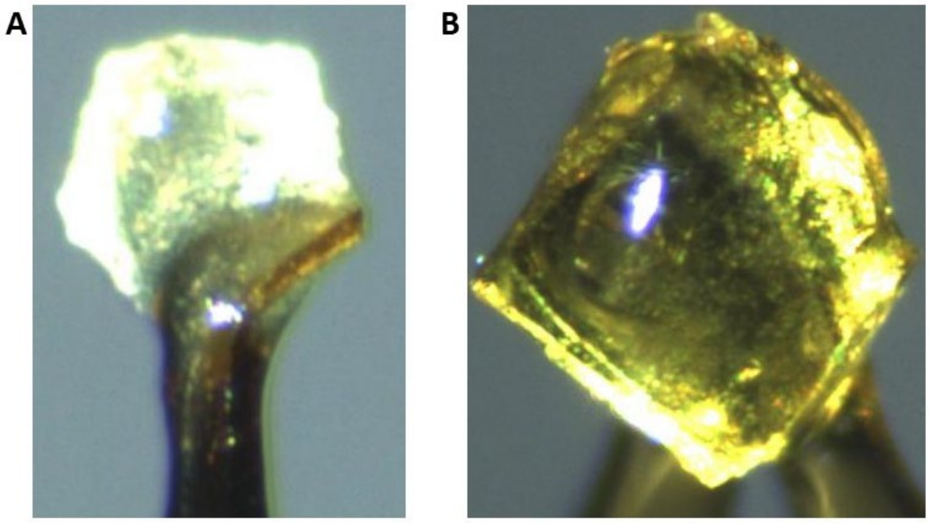

Figure S12. Images of A) Pt(IV)-biSi-1 and B) Pt(IV)-biSi-2 performed with a camera coupled to an X-Ray diffractometer.

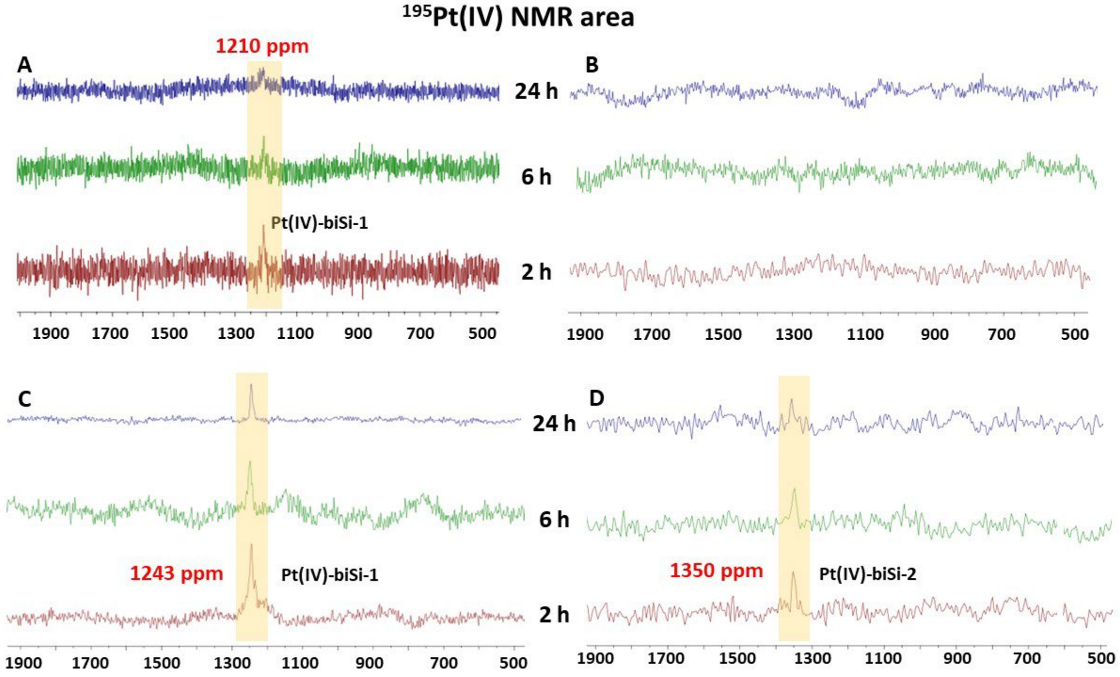

Figure S13. ^{195}Pt NMR spectra in the Pt(IV) region of the reduction kinetics at different times of: A) Pt(IV)-biSi-1 and AsA; B) Pt(IV)-biSi-2 and AsA; C) Pt(IV)-biSi-1 and GSH; D) Pt(IV)-biSi-2 and GSH.

A

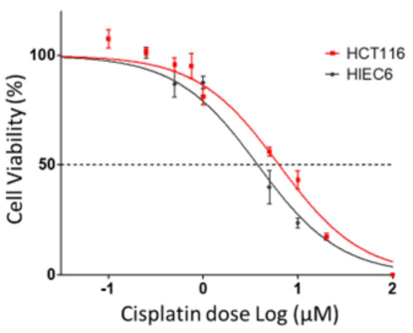

B

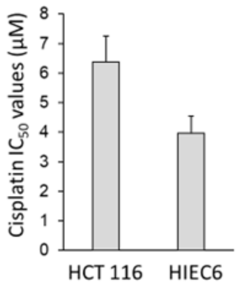

	Cisplatin (µM)	
	HCT116	HIEC6
IC_{50}	6.37	3.96
95% CI	5.03 to 8.07	3.07 to 5.11

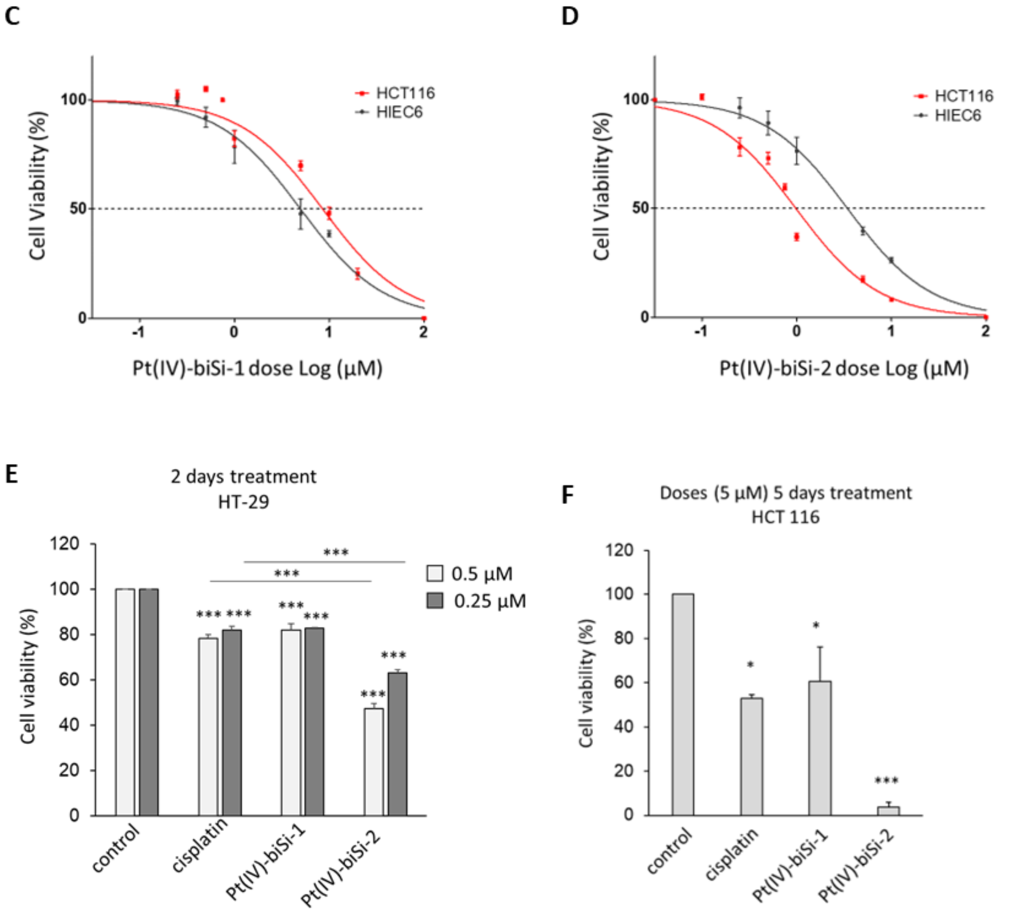

Figure S14. (A) Dose-response curve and (B) IC_{50} values for cisplatin on tumor (HCT 116) and healthy (HIEC6) human intestinal cell lines. (C-D) Dose-response curve for Pt(IV)-biSi-1 (C) and Pt(IV)-biSi-2 (D) on tumor (HCT 116) and healthy (HIEC6) human intestinal cell lines. In all panels HCT 116 (red) and HIEC6 (black) are shown. Cell viability was assessed after 48 h of treatment with Cisplatin (range 0–50 µM) by MTT assay. Cell viability is expressed as percentage. IC_{50} values are mean ± SEM of three independent experiments. 95% CI: confidence interval. Data are expressed as mean ± sem of three independent experiments. (E) MTT in HCT 116 cell after 5 days treatment with 5 µM cisplatin, Pt(IV)-biSi-1 or Pt(IV)-biSi-2. Cell viability is expressed as percentage. Values are means±SEM of three independent experiments.

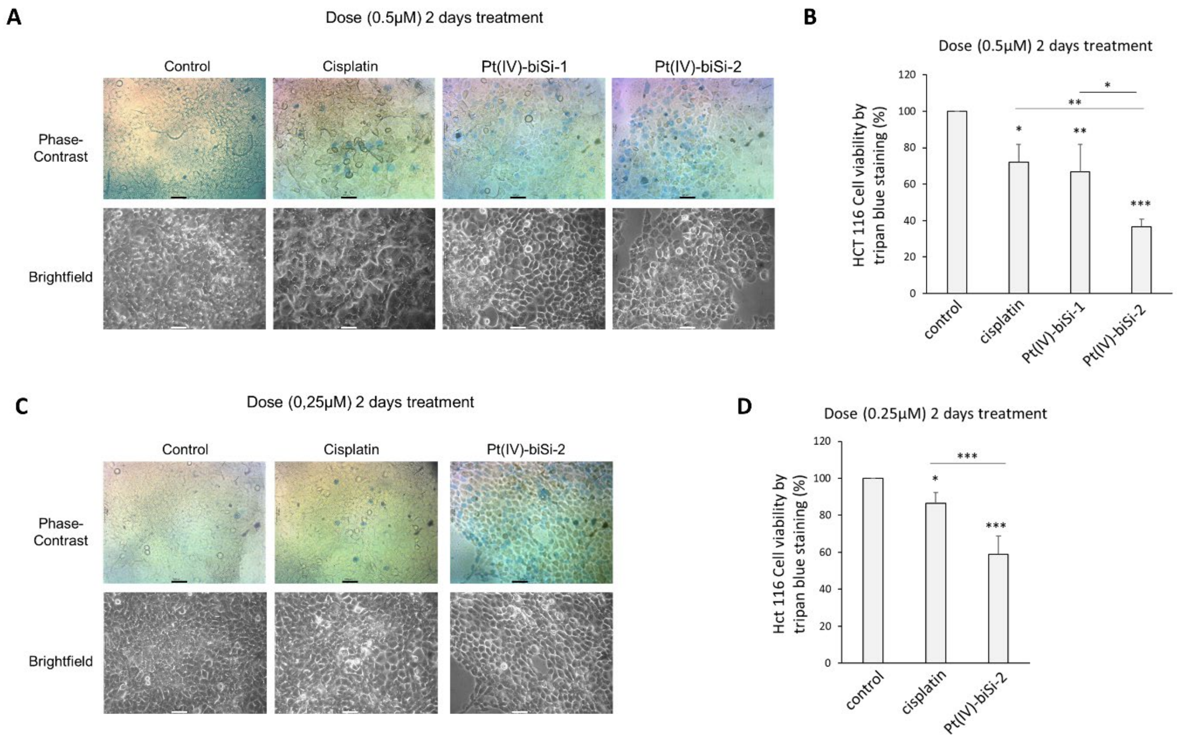

Figure S15. Viability estimation using exclusion of Trypan blue staining in viable cells. Representative pictures to show blue (dead) cells and brightfield to show the total. Quantification of three independent biological replicas and statistical analysis using ANOVA; *p<0.05; ** p<0.01; *** p<0.001.

A

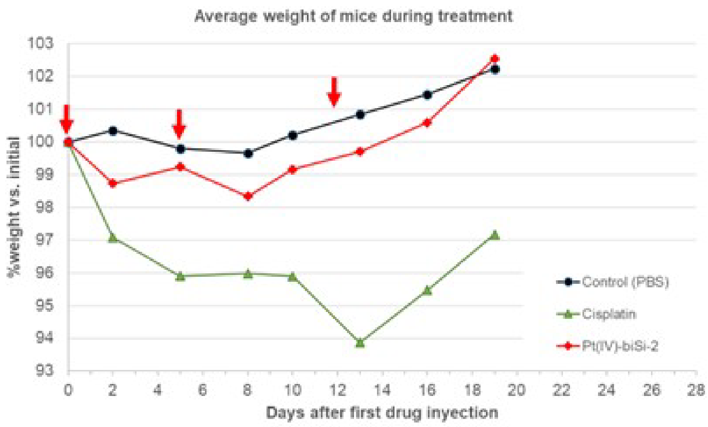

Nº animal with secondary effects (se) /Survival (sv)

Days after first treatment		0	2	5	8	10	13	16	19
Control (PBS)	se	0	0	0	0	0	0	0	0
	sv	*5*	*5*	*5*	*5*	*5*	*5*	*5*	*5*
Cisplatin	se	0	2	2	4	4	3	1	0
	sv	*5*	*5*	*5*	*4*	*4*	*3*	*2*	*2*
Pt(IV)-biSi-2	se	0	0	0	0	0	0	0	0
	sv	*5*	*5*	*5*	*5*	*5*	*5*	*5*	*5*

B

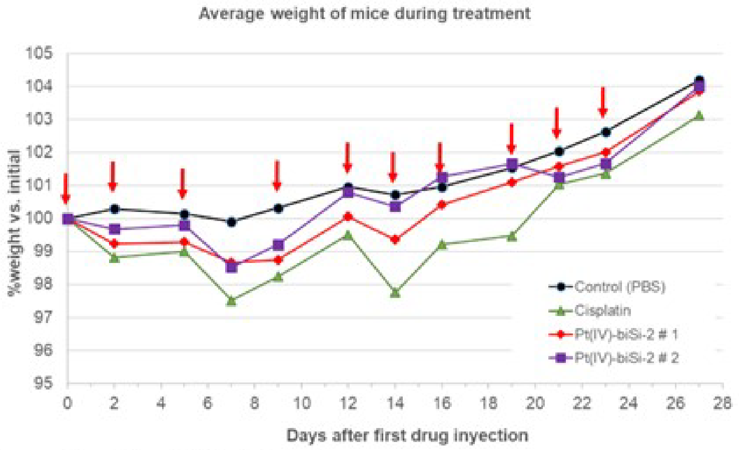

Nº animal with secondary effects (se) /Survival (sv)

Days after first treatment		0	2	5	7	9	12	14	16	19	21	23	27
Control (PBS)	se	0	0	0	0	0	0	0	0	0	0	0	0
	sv	*5*	*5*	*5*	*5*	*5*	*5*	*5*	*5*	*5*	*5*	*5*	*5*
Cisplatin	se	0	0	0	1	2	0	3	1	0	0	0	0
	sv	*5*	*5*	*5*	*5*	*5*	*5*	*4*	*4*	*4*	*4*	*4*	*4*
Pt(IV)-biSi-2 # 1	se	0	0	0	0	0	0	0	0	0	0	0	0
	sv	*6*	*6*	*6*	*6*	*6*	*6*	*6*	*6*	*6*	*6*	*6*	*6*
Pt(IV)-biSi-2 # 1	se	0	0	0	0	0	0	0	0	0	0	0	0
	sv	*4*	*4*	*4*	*4*	*4*	*4*	*4*	*4*	*4*	*4*	*4*	*4*

Figure S16. Side effects related to cisplatin and Pt(IV)-biSi-2 treatment at high (A) and low (B) concentrations in *in vivo* models. The graphs (top) shows the evolution of the average weight of the mice for each of the groups throughout the treatments (day of treatment indicated by red arrows). The tables (bottom) show for each of the treatments the number of animals that suffered some side effect related to the treatment (weight loss greater than 5% or abnormal postures), and the number of animals that survived. The drug concentration used depended on the results obtained in *in vitro* experiments. (A) Shows the results with acute treatment with 3 intraperitoneal injections using 4170 μM cisplatin (green triangles) or 1370 μM Pt(IV)-biSi-2 (red diamonds) are shown in (A). In (B) the results are shown for the chronic treatment with intraperitoneal injections using 417 μM of cisplatin (green triangles), 137 μM (red diamonds) or 417 μM (purple squares) of Pt(IV)-biSi-2. The tables show in blue background those days in which any of the above-mentioned events occurred.

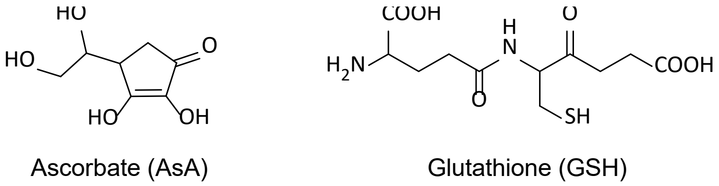

Scheme S1. Biomolecules used in this work to study the rate of reduction of the complexes Pt(IV)-biSi-1 and Pt(IV)-biSi-2.

Table S1. Chemical shift (δ) in ppm of the ^{1}H signals of the isopropylamine ligands of the complexes *cis*-[Pt(ipa)$_2$Cl$_2$] and iproplatin.

Complexes	*δ* (ppm) -NH$_2$	*δ* (ppm) -CH-	*δ* (ppm) –(CH$_3$)$_2$-
***cis*-[Pt(ipa)$_2$Cl$_2$]**	4.76	3.11	1.21
**iproplatin**	5.95	3.16	1.25
